# Sex differences in post-acute neurological sequelae of SARS-CoV-2 and symptom resolution in adults after coronavirus disease 2019 hospitalization: an international multi-centre prospective observational study

**DOI:** 10.1093/braincomms/fcae036

**Published:** 2024-02-09

**Authors:** Sung-Min Cho, Lavienraj Premraj, Denise Battaglini, Jonathon Paul Fanning, Jacky Suen, Gianluigi Li Bassi, John Fraser, Chiara Robba, Matthew Griffee, Tom Solomon, Malcolm G Semple, Kenneth Baillie, Louise Sigfrid, Janet T Scott, Barbara Wanjiru Citarella, Laura Merson, Rakesh C Arora, Glenn Whitman, David Thomson, Nicole White, ISARIC Clinical Characterization Group, Laurent Abel, Amal Abrous, Kamal Abu Jabal, Hiba Abu Zayyad, Younes Ait Tamlihat, Aliya Mohammed Alameen, Marta Alessi, Beatrice Alex, Kévin Alexandre, Adam Ali, Kazali Enagnon Alidjnou, Clotilde Allavena, Nathalie Allou, Claire Andréjak, Andrea Angheben, François Angoulvant, Séverine Ansart, Jean-Benoît Arlet, Elise Artaud-Macari, Jean Baptiste Assie, Johann Auchabie, Hugues Aumaitre, Adrien Auvet, Eyvind W Axelsen, Laurène Azemar, Cecile Azoulay, Benjamin Bach, Delphine Bachelet, Claudine Badr, Roar Bævre-Jensen, John Kenneth Baillie, Firouzé Bani-Sadr, Wendy S Barclay, Marie Bartoli, Joaquín Baruch, Romain Basmaci, Jules Bauer, Alexandra Bedossa, Husna Begum, Sylvie Behilill, Anna Beltrame, Marine Beluze, Nicolas Benech, Delphine Bergeaud, José Luis Bernal Sobrino, Giulia Bertoli, Simon Bessis, Sybille Bevilcaqua, Karine Bezulier, Krishna Bhavsar, Zeno Bisoffi, Laurent Bitker, Mathieu Blot, Laetitia Bodenes, Debby Bogaert, Anne-Hélène Boivin, Isabela Bolaños, Pierre-Adrien Bolze, François Bompart, Raphaël Borie, Elisabeth Botelho-Nevers, Lila Bouadma, Olivier Bouchaud, Sabelline Bouchez, Damien Bouhour, Kévin Bouiller, Laurence Bouillet, Camile Bouisse, Anne-Sophie Boureau, Maude Bouscambert, Aurore Bousquet, Marielle Boyer-Besseyre, Axelle Braconnier, Sonja Hjellegjerde Brunvoll, Marielle Buisson, Danilo Buonsenso, Aidan Burrell, Ingrid G Bustos, André Cabie, Eder Caceres, Cyril Cadoz, Jose Andres Calvache, Valentine Campana, Pauline Caraux-Paz, Nicolas Carlier, Thierry Carmoi, Marie-Christine Carret, Gail Carson, Maire-Laure Casanova, Guylaine Castor-Alexandre, François-Xavier Catherine, Paolo Cattaneo, Minerva Cervantes-Gonzalez, Anissa Chair, Catherine Chakveatze, Meera Chand, Jean-Marc Chapplain, Charlotte Charpentier, Julie Chas, Léo Chenard, Antoine Cheret, Thibault Chiarabini, Catherine Chirouze, Bernard Cholley, Marie-Charlotte Chopin, Yock Ping Chow, Barbara Wanjiru Citarella, Sara Clohisey, Gwenhaël Colin, Marie Connor, Anne Conrad, Graham S Cooke, Hugues Cordel, Andrea Cortegiani, Grégory Corvaisier, Camille Couffignal, Sandrine Couffin-Cadiergues, Roxane Courtois, Stéphanie Cousse, Juan Luis Cruz Bermúdez, Jaime Cruz Rojo, Elodie Curlier, Ana da Silva Filipe, Charlene Da Silveira, Andrew Dagens, John Arne Dahl, Jo Dalton, Etienne De Montmollin, Cristina De Rose, Thushan de Silva, Alexa Debard, Marie-Pierre Debray, Nathalie DeCastro, Romain Decours, Eve Defous, Isabelle Delacroix, Eric Delaveuve, Karen Delavigne, Christelle Delmas, Pierre Delobel, Elisa Demonchy, Emmanuelle Denis, Dominique Deplanque, Diane Descamps, Mathilde Desvallées, Alpha Diallo, Sylvain Diamantis, Fernanda Dias Da Silva, Kévin Didier, Jean-Luc Diehl, Jérôme Dimet, Vincent Dinot, Fara Diop, Alphonsine Diouf, Félix Djossou, Annemarie B Docherty, Christl A Donnelly, Céline Dorival, Eric D'Ortenzio, Nathalie Dournon, Thomas Drake, Amiel A Dror, Vincent Dubee, François Dubos, Alexandre Ducancelle, Susanne Dudman, Paul Dunand, Jake Dunning, Bertrand Dussol, Xavier Duval, Anne Margarita Dyrhol-Riise, Michael Edelstein, Linn Margrete Eggesbø, Mohammed El Sanharawi, Brigitte Elharrar, Merete Ellingjord-Dale, Philippine Eloy, Isabelle Enderle, Ilka Engelmann, Vincent Enouf, Olivier Epaulard, Hélène Esperou, Marina Esposito-Farese, Manuel Etienne, Mirjam Evers, Marc Fabre, Isabelle Fabre, Cameron J Fairfield, Karine Faure, Raphaël Favory, François-Xavier Ferrand, Eglantine Ferrand Devouge, Nicolas Ferriere, Céline Ficko, William Finlayson, Thomas Flament, Tom Fletcher, Aline-Marie Florence, Erwan Fourn, Robert A Fowler, Christophe Fraser, Stéphanie Fry, Valérie Gaborieau, Rostane Gaci, Jean-Charles Gagnard, Amandine Gagneux-Brunon, Sérgio Gaião, Linda Gail Skeie, Carrol Gamble, Noelia García Barrio, Esteban Garcia-Gallo, Denis Garot, Valérie Garrait, Anatoliy Gavrylov, Alexandre Gaymard, Eva Geraud, Louis Gerbaud Morlaes, Jade Ghosn, Tristan Gigante, Guillermo Giordano, Michelle Girvan, Valérie Gissot, Daniel Glikman, François Goehringer, Kyle Gomez, Marie Gominet, Yanay Gorelik, Isabelle Gorenne, Laure Goubert, Cécile Goujard, Tiphaine Goulenok, Pascal Granier, Christopher A Green, William Greenhalf, Segolène Greffe, Fiona Griffiths, Jérémie Guedj, Martin Guego, Romain Guery, Anne Guillaumot, Laurent Guilleminault, Thomas Guimard, Ali Hachemi, Nadir Hadri, Matthew Hall, Sophie Halpin, Rebecca Hamidfar, Bato Hammarström, Hayley Hardwick, Ewen M Harrison, Janet Harrison, Lars Heggelund, Ross Hendry, Maxime Hentzien, Diana Hernandez, Liv Hesstvedt, Rupert Higgins, Hikombo Hitoto, Antonia Ho, Alexandre Hoctin, Isabelle Hoffmann, Jan Cato Holter, Peter Horby, Ikram Houas, Jean-Sébastien Hulot, Samreen Ijaz, Patrick Imbert, Mariachiara Ippolito, Margaux Isnard, Mette Stausland Istre, Danielle Jaafar, Salma Jaafoura, Julien Jabot, Clare Jackson, Stéphane Jaureguiberry, Florence Jego, Synne Jenum, Silje Bakken Jørgensen, Cédric Joseph, Mercé Jourdain, Ouifiya Kafif, Florentia Kaguelidou, Sabina Kali, Deepjyoti Kalita, Karl Trygve Kalleberg, Christiana Kartsonaki, Seán Keating, Sadie Kelly, Kalynn Kennon, Younes Kerroumi, Antoine Khalil, Saye Khoo, Beathe Kiland Granerud, Anders Benjamin Kildal, Antoine Kimmoun, Eyrun Floerecke Kjetland Kjetland, Paul Klenerman, Gry Kloumann Bekken, Stephen R Knight, Arsène Kpangon, Oksana Kruglova, Galyna Kutsyna, Marie Lachatre, Marie Lacoste, Nadhem Lafhej, Marie Lagrange, Fabrice Laine, Olivier Lairez, Antonio Lalueza, Marc Lambert, Marie Langelot-Richard, Vincent Langlois, Cédric Laouénan, Samira Laribi, Delphine Lariviere, Stéphane Lasry, Odile Launay, Didier Laureillard, Yoan Lavie-Badie, Andy Law, Minh Le, Clément Le Bihan, Cyril Le Bris, Georges Le Falher, Lucie Le Fevre, Quentin Le Hingrat, Marion Le Maréchal, Soizic Le Mestre, Gwenaël Le Moal, Vincent Le Moing, Hervé Le Nagard, Jennifer Lee, Gary Leeming, Laurent Lefebvre, Bénédicte Lefebvre, Benjamin Lefèvre, Sylvie LeGac, Jean-Daniel Lelievre, Adrien Lemaignen, Véronique Lemee, Anthony Lemeur, Marc Leone, Quentin Lepiller, François-Xavier Lescure, Olivier Lesens, Mathieu Lesouhaitier, Sophie Letrou, Yves Levy, Bruno Levy, Claire Levy-Marchal, Erwan L'Her, Geoffrey Liegeon, Wei Shen Lim, Bruno Lina, Andreas Lind, Guillaume Lingas, Sylvie Lion-Daolio, Marine Livrozet, Paul Loubet, Bouchra Loufti, Guillame Louis, Jean Christophe Lucet, Carlos Lumbreras Bermejo, Miles Lunn, Liem Luong, Dominique Luton, Moïse Machado, Gabriel Macheda, Guillermo Maestro de la Calle, Rafael Mahieu, Sophie Mahy, Mylène Maillet, Thomas Maitre, Denis Malvy, Victoria Manda, Laurent Mandelbrot, Julie Mankikian, Aldric Manuel, Samuel Markowicz, John Marshall, Guillaume Martin-Blondel, Martin Martinot, Olga Martynenko, Mathieu Mattei, Laurence Maulin, Thierry Mazzoni, Colin McArthur, Sarah E McDonald, Kenneth A McLean, Cécile Mear-Passard, France Mentré, Alexander J Mentzer, Noémie Mercier, Emmanuelle Mercier, Antoine Merckx, Mayka Mergeay-Fabre, Laura Merson, Roberta Meta, Agnès Meybeck, Alison M Meynert, Vanina Meysonnier, Mehdi Mezidi, Céline Michelanglei, Isabelle Michelet, Sarah Moore, Shona C Moore, Lina Morales Cely, Lucia Moro, Hugo Mouquet, Clara Mouton Perrot, Julien Moyet, Jimmy Mullaert, Fredrik Müller, Karl Erik Müller, Marlène Murris, Srinivas Murthy, Nadège Neant, Anthony Nghi, Duc Nguyen, Alistair D Nichol, Mahdad Noursadeghi, Saad Nseir, Elsa Nyamankolly, Anders Benteson Nygaard, Piero L Olliaro, Wilna Oosthuyzen, Peter Openshaw, Claudia Milena Orozco-Chamorro, Paul Otiku, Nadia Ouamara, Rachida Ouissa, Eric Oziol, Maïder Pagadoy, Justine Pages, Massimo Palmarini, Prasan Kumar Panda, Nathalie Pansu, Aurélie Papadopoulos, Rachael Parke, Jérémie Pasquier, Bruno Pastene, Christelle Paul, William A Paxton, Jean-François Payen, Miguel Pedrera Jiménez, Florent Peelman, Nathan Peiffer-Smadja, Vincent Peigne, Daniel Perez, Thomas Perpoint, Vincent Pestre, Ventzislava Petrov-Sanchez, Frank Olav Pettersen, Gilles Peytavin, Walter Picard, Olivier Picone, Lionel Piroth, Chiara Piubelli, Riinu Pius, Laurent Plantier, Julien Poissy, Ryadh Pokeerbux, Georgios Pollakis, Diane Ponscarme, Sébastien Preau, Mark G Pritchard, Víctor Quirós González, Else Quist-Paulsen, Christian Rabaud, Marie Rafiq, Blandine Rammaert, Christophe Rapp, Stanislas Rebaudet, Sarah Redl, Dag Henrik Reikvam, Martine Remy, Anne-Sophie Resseguier, Matthieu Revest, Luis Felipe Reyes, Antonia Ricchiuto, Laurent Richier, Patrick Rispal, Karine Risso, Stephanie Roberts, David L Robertson, Olivier Robineau, Paola Rodari, Pierre-Marie Roger, Amanda Rojek, Roberto Roncon-Albuquerque, Mélanie Roriz, Manuel Rosa-Calatrava, Andrea Rossanese, Patrick Rossignol, Carine Roy, Benoît Roze, Clark D Russell, Aleksander Rygh Holten, Charlotte Salmon Gandonniere, Hélène Salvator, Olivier Sanchez, Vanessa Sancho-Shimizu, Pierre-François Sandrine, Oana Sandulescu, Benjamine Sarton, Egle Saviciute, Arnaud Scherpereel, Marion Schneider, Janet T Scott, James Scott-Brown, Nicholas Sedillot, Malcolm G Semple, Eric Senneville, Pablo Serrano Balazote, Catherine A Shaw, Victoria Shaw, Girish Sindhwani, Nassima Si Mohammed, Jeanne Sibiude, Louise Sigfrid, Dario Sinatti, Vegard Skogen, Sue Smith, Lene Bergendal Solberg, Tom Solomon, Agnès Sommet, Arne Søraas, Albert Sotto, Edouard Soum, Elisabetta Spinuzza, Shiranee Sriskandan, Sarah Stabler, Trude Steinsvik, Birgitte Stiksrud, Adrian Streinu-Cercel, Anca Streinu-Cercel, David Stuart, Richa Su, Charlotte Summers, Lysa Tagherset, Renaud Tamisier, Coralie Tardivon, Pierre Tattevin, Marie-Capucine Tellier, François Téoulé, Olivier Terrier, Nicolas Terzi, Vincent Thibault, Simon-Djamel Thiberville, Benoît Thill, Emma C Thomson, Mathew Thorpe, Ryan S Thwaites, Vadim Tieroshyn, Jean-François Timsit, Noémie Tissot, Kristian Tonby, Cécile Tromeur, Tiffany Trouillon, Jeanne Truong, Christelle Tual, Sarah Tubiana, Jean-Marie Turmel, Lance C W Turtle, Anders Tveita, Timothy M Uyeki, Piero Valentini, Sylvie Van Der Werf, Noémie Vanel, Charline Vauchy, Aurélie Veislinger, Benoit Visseaux, Fanny Vuotto, Steve Webb, Jia Wei, Murray Wham, Paul Henri Wicky, Aurélie Wiedemann, Natalie Wright, Yazdan Yazdanpanah, Cécile Yelnik, Hodane Yonis, Marion Zabbe, Maria Zambon, David Zucman

**Affiliations:** Neuroscience Critical Care Division, Department of Neurology, Johns Hopkins University School of Medicine, Baltimore, MD 21278, USA; Neuroscience Critical Care Division, Department of Surgery, Johns Hopkins University School of Medicine, Baltimore, MD 21278, USA; Neuroscience Critical Care Division, Department of Anaesthesiology and Critical Care Medicine, Johns Hopkins University School of Medicine, Baltimore, MD 21278, USA; Australian Centre for Health Services Innovation and Centre for Healthcare Transformation, School of Public Health and Social Work, Queensland University of Technology, Kelvin Grove 4059, Australia; Griffith University School of Medicine, Gold Coast 4215, Australia; Critical Care Research Group, The Prince Charles Hospital, Brisbane 4032, Australia; Department of Surgical Science and Integrated Diagnostic, San Martino Policlinico Hospital, IRCCS for Oncology and Neuroscience, University of Genoa, Genoa 16132, Italy; Department of Medicine, University of Barcelona, Barcelona 08036, Spain; Critical Care Research Group, The Prince Charles Hospital, Brisbane 4032, Australia; Faculty of Medicine, University of Queensland, Brisbane 4006, Australia; Critical Care Research Group, The Prince Charles Hospital, Brisbane 4032, Australia; Faculty of Medicine, University of Queensland, Brisbane 4006, Australia; Australian Centre for Health Services Innovation and Centre for Healthcare Transformation, School of Public Health and Social Work, Queensland University of Technology, Kelvin Grove 4059, Australia; Critical Care Research Group, The Prince Charles Hospital, Brisbane 4032, Australia; Faculty of Medicine, University of Queensland, Brisbane 4006, Australia; Institut d’Investigacions Biomediques August Pi I Sunyer, Barcelona 08036, Spain; Australian Centre for Health Services Innovation and Centre for Healthcare Transformation, School of Public Health and Social Work, Queensland University of Technology, Kelvin Grove 4059, Australia; Critical Care Research Group, The Prince Charles Hospital, Brisbane 4032, Australia; Faculty of Medicine, University of Queensland, Brisbane 4006, Australia; St Andrew’s War Memorial Hospital, UnitingCare, Spring Hill 4000, Australia; Department of Surgical Science and Integrated Diagnostic, San Martino Policlinico Hospital, IRCCS for Oncology and Neuroscience, University of Genoa, Genoa 16132, Italy; Department of Anesthesiology, University of Utah, Salt Lake City, UT 84132, USA; Brain Infections Group, Institute of Infection and Global Health, University of Liverpool, Liverpool, L3 5TR, UK; Department of Neuroscience, University of Liverpool, Liverpool, L3 5TR, UK; Walton Centre NHS Foundation Trust, Liverpool, L9 7LJ, UK; Child Health and Outbreak Medicine, University of Liverpool, Liverpool, L3 5TR, UK; Experimental Medicine, University of Edinburgh, Edinburgh, EH4 2XU, UK; Centre for Tropical Medicine and Global Health, University of Oxford, Oxford, OX3 7LG, UK; Infectious Disease, University of Glasgow, Glasgow, G12 8QQ, UK; International Severe Acute Respiratory and emerging Infections Consortium (ISARIC), Pandemic Sciences Institute, University of Oxford, Oxford, OX1 2JD, UK; Institut d’Investigacions Biomediques August Pi I Sunyer, Barcelona 08036, Spain; Department of Surgery, University Hospitals/Case Western Reserve University, Cleveland, OH 44106, USA; Department of Surgery, Johns Hopkins University School of Medicine, Baltimore, MD 21278, USA; Department of Anaesthesia and Peri-operative Medicine, University of Cape Town, Cape Town 7700, South Africa; Division of Critical Care, Groote Schuur Hospital, Cape Town 7925, South Africa; Australian Centre for Health Services Innovation and Centre for Healthcare Transformation, School of Public Health and Social Work, Queensland University of Technology, Kelvin Grove 4059, Australia

**Keywords:** COVID-19, SARS-CoV-2, neurological complication, neurological long COVID, post-acute neurological sequelae of SARS-CoV-2 (PANSC)

## Abstract

Although it is known that coronavirus disease 2019 can present with a range of neurological manifestations and in-hospital complications, sparse data exist on whether these initial neurological symptoms of coronavirus disease 2019 are closely associated with post-acute neurological sequelae of SARS-CoV-2 (severe acute respiratory syndrome coronavirus 2; PANSC) and whether female versus male sex impacts symptom resolution. In this international, multi-centre, prospective, observational study across 407 sites from 15 countries (30 January 2020 to 30 April 2022), we report the prevalence and risk factors of PANSC among hospitalized adults and investigate the differences between males and females on neurological symptom resolution over time. PANSC symptoms included altered consciousness/confusion, fatigue/malaise, anosmia, dysgeusia and muscle ache/joint pain, on which information was collected at index hospitalization and during follow-up assessments. The analysis considered a time to the resolution of individual and all neurological symptoms. The resulting times were modelled by Weibull regression, assuming mixed-case interval censoring, with sex and age included as covariates. The model results were summarized as cumulative probability functions and age-adjusted and sex-adjusted median times to resolution. We included 6862 hospitalized adults with coronavirus disease 2019, who had follow-up assessments. The median age of the participants was 57 years (39.2% females). Males and females had similar baseline characteristics, except that more males (versus females) were admitted to the intensive care unit (30.5 versus 20.3%) and received mechanical ventilation (17.2 versus 11.8%). Approximately 70% of patients had multiple neurological symptoms at the first follow-up (median = 102 days). Fatigue (49.9%) and myalgia/arthralgia (45.2%) were the most prevalent symptoms of PANSC at the initial follow-up. The reported prevalence in females was generally higher (versus males) for all symptoms. At 12 months, anosmia and dysgeusia were resolved in most patients, although fatigue, altered consciousness and myalgia remained unresolved in >10% of the cohort. Females had a longer time to the resolution (5.2 versus 3.4 months) of neurological symptoms at follow-up for those with more than one neurological symptom. In the multivariable analysis, males were associated with a shorter time to the resolution of symptoms (hazard ratio = 1.53; 95% confidence interval = 1.39–1.69). Intensive care unit admission was associated with a longer time to the resolution of symptoms (hazard ratio = 0.68; 95% confidence interval = 0.60–0.77). Post-discharge stroke was uncommon (0.3% in females and 0.5% in males). Despite the methodological challenges involved in the collection of survey data, this international multi-centre prospective cohort study demonstrated that PANSC following index hospitalization was high. Symptom prevalence was higher and took longer to resolve in females than in males. This supported the fact that while males were sicker during acute illness, females were disproportionately affected by PANSC.

## Teaser

The authors report that post-acute neurological sequelae of SARS-CoV-2 (PANSC) following index hospitalization is high in 6862 hospitalized adults with coronavirus disease 2019. Symptom prevalence is higher and takes longer to resolve in females than in males, showing that while males are sicker during acute illness, females are disproportionately affected by PANSC.

## Graphical Abstract

**Graphical Abstract:**
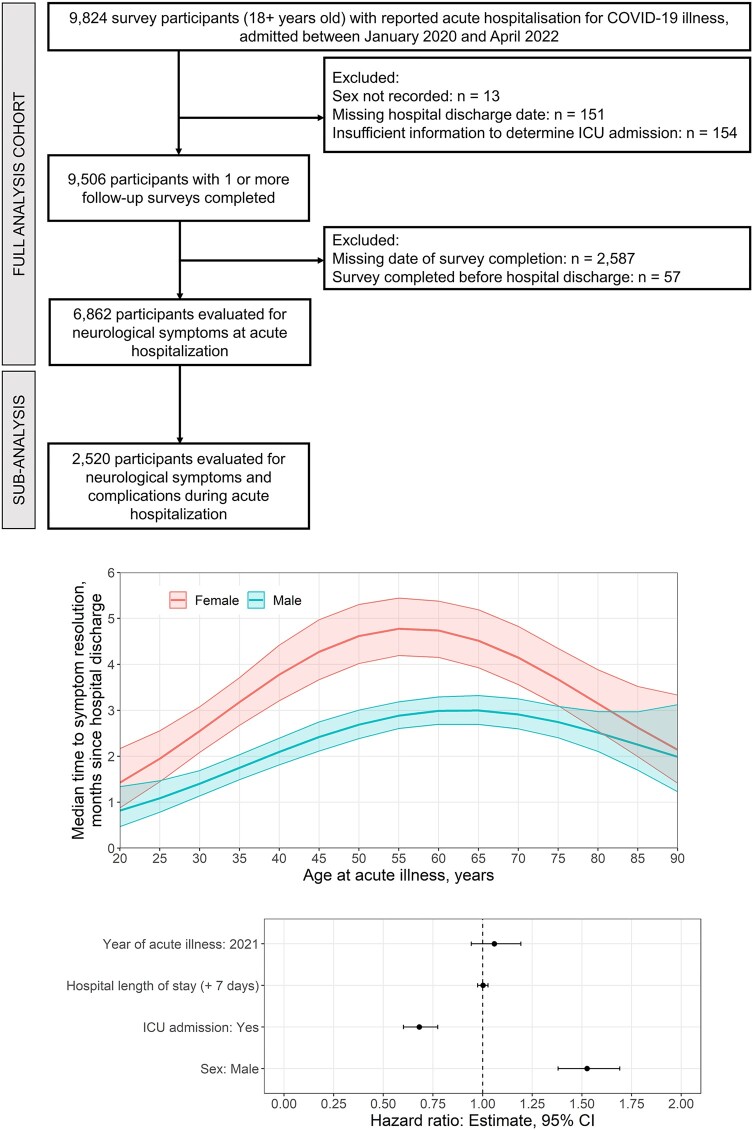

## Introduction

Despite multifaceted global efforts in public health measures such as vaccination and medical treatments that have improved the overall outcome of coronavirus disease 2019 (COVID-19), the rapid evolution of severe acute respiratory syndrome coronavirus 2 (SARS-CoV-2) with multiple variants, coupled with complex social, economic and political ramifications, has hindered a return to pre-pandemic normalcy. Moreover, reports on post-acute sequelae of SARS-CoV-2 infection (PANSC or long COVID) have accumulated after the declaration of the COVID-19 pandemic in March 2020. COVID-19 can cause long-term symptoms that can persist for months or even years after acute COVID-19 infection.^1,2^ The intensity of these symptoms can vary in severity from mild to debilitating. Among long COVID symptoms, neurological symptoms are of particular public health concern and societal burden, which are increasing in prevalence over time as millions of people are infected with COVID-19.^3,4^

Although it is well established that acute COVID-19 infection can present with neurological manifestations and in-hospital complications,^5-8^ sparse data exist regarding whether these initial neurological symptoms and presentations of COVID-19 are closely associated with post-acute neurological sequelae of SARS-CoV-2 (PANSC). Despite many reports on the incidence and risk factors of long COVID over the last few years,^9-11^ high-quality evidence focused on ‘neurological’ long COVID is currently limited. Therefore, a robust large-scale international multi-centre research is necessary to study the prevalence, risk factors and trajectory of long-term PANSC after acute COVID-19 infection, especially for those with COVID-19 hospitalization. Prior studies reported a significant association between female sex and long COVID.^10,12^ However, most studies did not and do not report separate data between sexes on neurological long COVID. Therefore, the primary aim of this study was to describe the prevalence and risk factors of persistent long-term PANSC and post-discharge neurological complications of COVID-19 among hospitalized adults from a large multi-centre COVID-19 registry, the International Severe Acute Respiratory and emerging Infection Consortium (ISARIC) COVID-19 database; a secondary aim was to investigate the impact of female sex on long-term PANSC resolution over time.

## Materials and methods

The original study protocol was approved by the World Health Organization Ethics Review Committee, and local ethics approval was obtained for each participating country and site according to local requirements. Patient consent was obtained for a follow-up survey. De-identified data were submitted to the ISARIC database by direct entry to Research Electronic Data Capture (REDCap, version 8.11.11, Vanderbilt University, Nashville, TN, USA), hosted by the University of Oxford or by secure file transfer when locally managed data collection systems were used. All data submitted to the ISARIC data platform were harmonized to the CDISC SDTM standard (Study Data Tabulation Model; version 1.7, Clinical Data Interchange Standards Consortium, Austin, TX, USA). A full description of data collection and curation methods is available.^13^

### Study design

Data from a multi-centre, international observational study were analysed to ascertain the prevalence and characteristics of long-term PANSC and neurological complications after a discharge from acute COVID-19 hospitalization. Patient data were collected according to the ISARIC COVID-19 Follow-Up Study Protocol, a prospective study of COVID-19 patients to assess frequency and risk factors for long-term health and psychosocial consequences of COVID-19.^14,15^ The original ISARIC registry included hospitalized patients with COVID-19 symptoms and laboratory-confirmed SARS-CoV-2. A subset of hospitalized patients with COVID-19 were assessed using received standardized case report forms (CRFs) post-discharge (ISARIC Global Follow-Up Tier 1 Survey: CRFs available in Appendix I) to do a follow-up and collect long-term physical and psychosocial sequelae. We analysed survey items related to PANSC and post-discharge neurological complications.

Available data are described in the ISARIC Global follow-up protocol and CRFs (https://isaric.org/research/covid-19-clinical-research-resources/covid-19-long-term-follow-up-study-old/).^15,16^ The hospitalization data including presentation and in-hospital neurological complications^8^ were linked with the follow-up survey data by a unique participant identifier. Serial follow-up was continued every 3 months post-discharge for up to 12 months. Individuals were recruited for the follow-up study at post-COVID outpatient units. All presenting individuals were invited to join the study. Patients are referred to these units from various sources, including emergency departments, admission wards and family doctors in the recruiting regions. Although all patients admitted with acute COVID-19 during the recruitment period were invited to participate in follow-up, only a random sampling of patients were invited due to resource constraints.

### Cohorts

**Figure 1:**
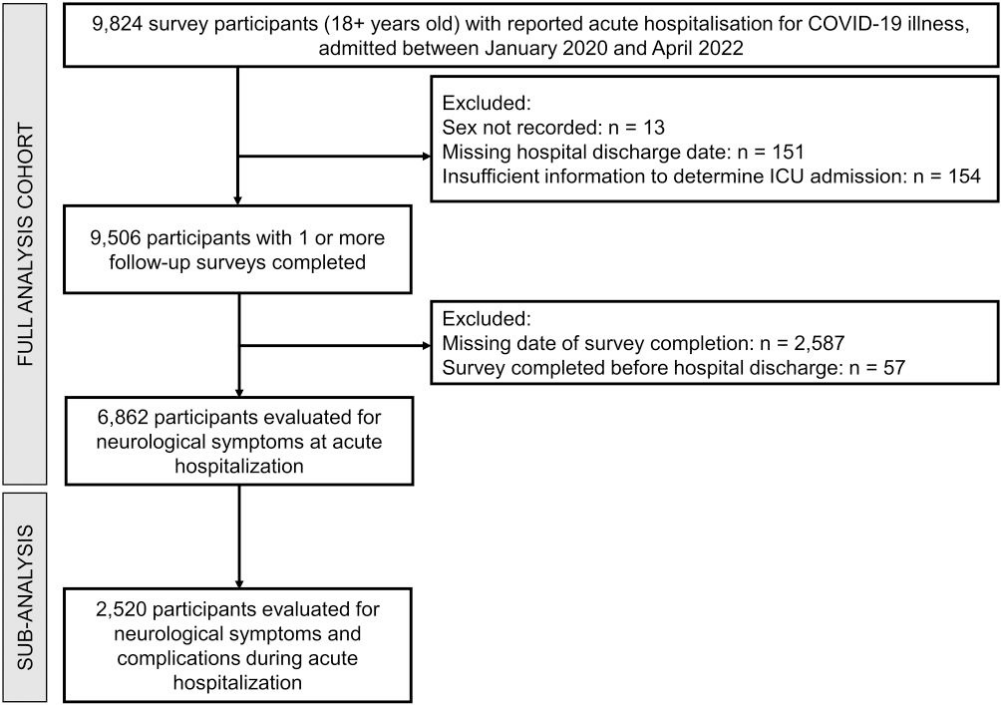
**A study flow diagram.** A flow diagram of the study cohort who participated in a long-term outcome survey.

The study cohort for analysis included all patients aged 18 years or over enrolled in the ISARIC COVID-19 Follow-Up Study with confirmed COVID-19 infection who were hospitalized with symptomatic COVID-19 between 30 January 2020 and 30 March 2022. We excluded patients in whom information on hospital admission and discharge dates, sex, intensive care unit (ICU) admission, neurological manifestations/complications and neuropsychiatric follow-up data was missing (Fig. 1).

Participants were followed up regularly for up to a year post-discharge. Eligible participants completed one or more surveys, with the first survey completed at least 1-month post-discharge from their hospitalization (data completeness available in Supplementary Table 1). The Tier 1 initial survey was used at the initial follow-up time point (at 3 months post-discharge) and repeated every 3–6 months thereafter. The survey was designed for assessment via patient self-completion, administered via an online link or as a paper form, or by a clinician via in-clinic or telephone assessments during in-person follow-up.

### Definitions

Neurological symptoms, as collected at index hospitalization and during follow-up assessments, included altered consciousness/confusion, fatigue/malaise, anosmia, dysgeusia and muscle aches/joint pain. Long-term complications after discharge from COVID-19 admission included stroke/transient ischaemic attack (TIA), deep vein thrombosis (DVT), pulmonary embolism (PE), heart attack and kidney problems.

### Outcomes

The primary outcome was the presence of PANSC and post-discharge stroke/TIA at each follow-up time. The secondary outcome was PANSC symptom resolution, accounting for associations with known risk factors with sex (female versus male) as a covariate of interest.

### Statistical analysis

Respondent characteristics collected at initial acute COVID-19 hospitalization were summarized for the full analysis cohort and by sex. All continuous variables were summarized as medians and quartiles; categorical variables were summarized as frequencies and percentages. Characteristics covered respondent demographics, comorbidities, neurological symptoms reported at admission, in-hospital neurological complications, ICU admission, mechanical ventilation, extracorporeal membrane oxygenation (ECMO) and hospital length of stay. The reported prevalence of neurological symptoms was estimated at index hospitalization and initial survey follow-up. Overall and sex-specific prevalences were estimated by crude and age-adjusted rates per 100 hospital discharges with 95% confidence intervals (CIs).

The analysis of long-term neurological outcomes considered the time to the resolution of individual neurological symptoms and the resolution of all neurological symptoms present at initial hospitalization. Two definitions for symptom resolution were considered based on longitudinal survey responses. For the primary analysis, symptom resolution was defined as the first survey date where a participant reported the symptom as absent. As a sensitivity analysis, symptom resolution was defined by participant responses at their last known date of survey follow-up. Resulting times were modelled by Weibull regression, assuming mixed-case interval censoring, with sex and age included as covariates. Model results were summarized as cumulative probability functions and age-adjusted and sex-adjusted median times to resolution. Additional multivariable analyses considered ICU status, year of acute illness, hospital length of stay and reported in-hospital neurological complications. Further details on the methods are provided in Supplementary File 1.

## Results

**Figure 2:**
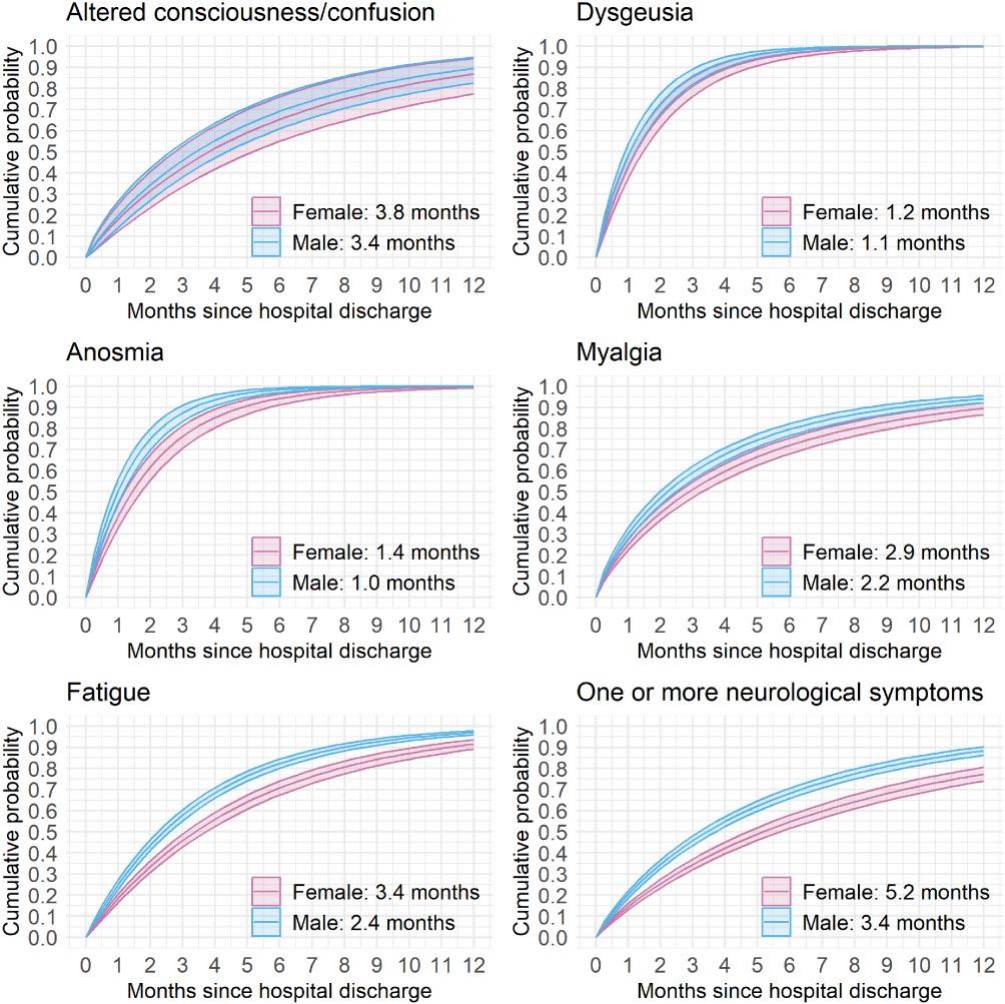
**Symptom resolution probability.** Cumulative probability functions [estimate (95% CI)] to neurological symptom resolution stratified by sex (female = pink, male = blue; adjusted for age = 60 years). Median times for symptom resolution for the corresponding sex are annotated (also see Supplementary Table 5). Sensitivity analysis results are provided in Supplementary Fig. 8.

The initial survey cohort included 9824 adult patients hospitalized due to COVID-19 from 407 sites and 15 countries (Fig. 1). Of these, 6862 participants satisfied the inclusion criteria as a final study cohort. Initial follow-up assessment occurred at 102 days [median, interquartile range (IQR) = 77–183 days] following discharge from the index hospitalization. Supplementary Tables 1 and 2 and Figs. 1 and 2 further describe data completeness, survey timing, frequency and response rate. For our analysis of time to symptom resolution, available participant numbers were as follows: 2971 (0–3 months), 3188 (3–6 months), 1514 (6–9 months) and 2792 (9+ months). The median time to participation loss to follow-up was 6 months (Supplementary Fig. 8).

### Baseline characteristics

Overall, 6862 participants were analysed (39.2% females) with a median age of 57 years (IQR = 46–67 years; Table 1). Most participants were recruited from high-income countries, with European and Asian regions accounting for 98.2% of the study cohort. A geographic breakdown and summary of comorbidities are given in Supplementary Table 3. Males and females had similar baseline characteristics, though neurological symptoms at hospital admission for acute COVID-19 were marginally more common in females than in males (Table 1). Fatigue (54.9%) and myalgia (34.8%) were the most frequently reported neurological symptoms at hospital admission. Fatigue was most frequently reported together with myalgia, dysgeusia and anosmia (Supplementary Fig. 3 and Supplementary Fig. 4). Neurological complications of COVID-19 during hospitalization were uncommon (1.1%); stroke was more common in males than in females (Supplementary Table 4). Of hospitalized patients, 26.5% were admitted to ICU, 15.1% received mechanical ventilation and none received ECMO. More males than females were admitted to the ICU (30.5 versus 20.3%) and received mechanical ventilation (17.2 versus 11.8%; Table 1).

**Table 1:** Baseline demographic and index hospitalization characteristics

Characteristic	Full cohort (*n* = 6862)	Female (*n* = 2691)	Male (*n* = 4171)
Age, years	57 (46–67)	56 (45–66)	58 (47–67)
High-income country	5169 (75.3)	2079 (77.3)	3090 (74.1)
Geographic region^a^			
Africa	77 (1.1)	46 (1.7)	31 (0.7)
Asia	1650 (24.0)	573 (21.3	1077 (25.8)
Europe	5092 (74.2)	2055 (76.4)	3037 (72.8)
Latin America and the Caribbean	43 (0.6)	17 (0.6)	26 (0.6)
Comorbidities^b^			
Chronic cardiac disease^c^	705 (10.3)	237 (8.8)	468 (11.3)
Chronic kidney disease^d^	319 (4.7)	102 (3.8)	217 (5.2)
Chronic neurological disorder^e^	240 (3.5)	124 (4.6)	116 (2.8)
Diabetes mellitus	1142 (25.1)	387 (22.2)	755 (26.9)
Obesity	529 (19.6)	255 (23.4)	274 (17.0)
Neurological complications during hospitalization^f^			
Meningitis/encephalitis^g^	8 (0.3)	3 (0.3)	5 (0.3)
Seizures^h^	9 (0.4)	6 (0.6)	3 (0.2)
Stroke^i^	12 (0.5)	3 (0.3)	9 (0.6)
One or more complications	27 (1.1)	11 (1.0)	16 (1.1)
Neurological symptoms at hospital admission			
Altered consciousness	467 (7.1)	17 (6.8)	290 (7.2)
Fatigue	3607 (54.9)	1485 (57.4)	2122 (53.2)
Anosmia	1108 (18.3)	462 (19.4)	646 (17.5)
Dysgeusia	1241 (20.4)	523 (21.9)	718 (19.5)
Myalgia	2253 (34.8)	989 (38.6)	1264 (32.3)
Seizure	33 (0.7)	13 (0.7)	20 (0.7)
One or more symptoms	4565 (66.5)	1854 (68.9)	2711 (65.0)
COVID-19 index hospitalization information			
ICU admission	1818 (26.5)	547 (20.3)	1271 (30.5)
Mechanically ventilated	993 (15.1)	301 (11.8)	692 (17.2)
ECMO	0 (0.0)	0 (0.0)	0 (0.0)
Antivirals	887 (17.3)	332 (16.6)	555 (17.8)
Corticosteroids	1314 (32.1)	501 (31.2)	813 (32.7)
Hospital length of stay, days	8 (5–13)	7 (4–11)	8 (5–14)

ICU, intensive care unit; ECMO, extracorporeal membrane oxygenation. ^a^See Supplementary Table 3 for a detailed breakdown by geographic sub-region. ^b^Comorbidities are reported at initial hospitalization; see Supplementary Table 3 for additional comorbidities. ^c^Chronic cardiac disease: any of coronary artery disease, heart failure, congenital heart disease, cardiomyopathy, or rheumatic heart disease (not hypertension). ^d^Chronic kidney disease: chronic estimated glomerular filtration rate <60 ml/min/1.73 m^2^ or history of kidney transplantation. ^e^Chronic neurologic disorder: any of cerebral palsy, multiple sclerosis, motor neuron disease, muscular dystrophy, myasthenia gravis, Parkinson’s disease, stroke and severe learning difficulty. ^f^Subset of respondents evaluated for in-hospital complications (*n* = 2520; Fig. 1). ^g^Inclusive of all seizures during hospitalization regardless of cause. ^h^Clinical diagnosis of stroke with or without radiological findings. ^i^Inflammation of the meninges or the brain parenchyma diagnosed clinically, radiologically, or microbiologically.

### Post-discharge stroke

Approximately 1.1% of male and female participants experience one or more neurological complications during hospitalization (Table 1). During survey follow-up, stroke/TIA was uncommon: 0.3% in females and 0.4% in males. Other complications including DVT, PE, heart attack and kidney problems were infrequent in both males and females (0–2% range).

### PANSC

Approximately 70% of patients had more than one neurological symptom upon initial follow-up (median follow-up time = 99 days, IQR = 79–177 days; Supplementary Table 2). Fatigue (49.9%; 95% CI = 47.6–52.4; age-standardized: 47.9; 45.1–50.9 cases per 100 discharges) and myalgia/arthralgia (45.2%; 95% CI = 42.4–48.1; age-standardized: 43.8; 40.8–47.0 cases per 100 discharges) were the most frequently reported symptoms of PANSC. Seventy-five per cent of female participants reported one or more persistent neurological symptoms at initial follow-up (95% CI = 71.4–79.5; age-standardized: 73.2; 67.5–79.5 cases per 100 discharges). Reported prevalence in females was generally higher than in males for all PANSC symptoms at initial follow-up (Table 2). Dizziness, visual problems and sleeping problems were reported more frequently by females than males (Supplementary Fig. 5).

**Table 2:** The observed prevalence of neurological symptoms at initial survey follow-up, stratified by the presence or absence at acute COVID-19 hospitalization

	Full cohort		Female		Male	
	Present	Absent	Present	Absent	Present	Absent
Altered consciousness/confusion						
Reported cases	195/368	1112/4398	84/143	504/1717	111/225	608/2681
Crude	53.0 (45.8–61.0)	25.3 (23.8–26.8)	58.7 (46.9–72.7)	29.4 (26.8–32.0)	49.3 (40.6–59.4)	22.7 (20.9–24.6)
Age-standardized	41.7 (31.7–55.5)	24.3 (22.5–26.5)	39.2 (21.6–79.4)	28.7 (25.1–33.0)	41.7 (30.8–57.2)	21.9 (19.8–24.3)
Fatigue						
Reported cases	1651/3306	993/2862	772/1339	419/1057	879/1967	574/1805
Crude	49.9 (47.6–52.4)	34.7 (32.6–36.9)	57.7 (53.7–61.9)	39.6 (35.9–43.6)	44.7 (41.8–47.7)	31.8 (29.3–34.5)
Age-standardized	47.9 (45.1–50.9)	34.5 (31.6–37.7)	55.7 (50.5–61.7)	39.1 (33.8–45.4)	42.6 (39.5–46.1)	32.0 (28.7–35.8)
Anosmia						
Reported cases	197/1073	293/4759	108/448	157/1849	89/625	136/2910
Crude	18.4 (15.9–21.1)	6.2 (5.5–6.9)	24.1 (19.8–29.1)	8.5 (7.2–9.9)	14.2 (11.4–17.5)	4.7 (3.9–5.5)
Age-standardized	17.7 (15.3–20.8)	5.7 (4.9–6.7)	22.8 (18.6–31.9)	7.6 (6.1–9.6)	13.8 (11.1–17.4)	4.4 (3.6–5.6)
Dysgeusia						
Reported cases	182/1198	277/4627	95/504	149/1797	87/694	128/2830
Crude	15.2 (13.1–17.6)	6.0 (5.3–6.7)	18.8 (15.3–23.0)	8.3 (7.0–9.7)	12.5 (10.0–15.5)	4.5 (3.8–5.4)
Age-standardized	14.5 (12.4–17.1)	5.8 (4.9–6.9)	17.9 (14.4–24.9)	7.7 (6.1–9.8)	12.1 (9.6–15.3)	4.5 (3.6–5.9)
Myalgia						
Reported cases	1000/2213	1124/4110	495/966	474/1531	505/1247	650/2579
Crude	45.2 (42.4–48.1)	27.3 (25.8–29.0)	51.2 (46.8–56.0)	31.0 (28.2–33.9)	40.5 (37.0–44.2)	25.2 (23.3–27.2)
Age-standardized	43.8 (40.8–47.0)	26.8 (24.8–29.0)	50.6 (45.3–56.7)	29.6 (26.1–33.8)	38.9 (35.3–42.9)	25.2 (22.8–27.8)
Seizures						
Reported cases	0/23 (0.0)	24/3060 (0.8)	0/9 (0.0)	11/1203 (0.9)	0/14 (0.0)	13/1857 (0.7)
Crude		0.8 (0.5–1.2)		0.9 (0.5–1.6)		0.7 (0.4–1.2)
Age-standardized		1.1 (0.5–2.2)		1.8 (0.5–4.8)		0.7 (0.3–1.9)
One or more neurological symptoms						
Reported cases	3005/4366	1311/2496	1327/1761	534/930	1678/2605	777/1566
Crude	68.8 (66.4–71.3)	52.5 (49.7–55.4)	75.4 (71.4–79.5)	57.4 (52.7–62.5)	64.4 (61.4–67.6)	49.6 (46.2–53.2)
Age-standardized	67.3 (64.1–70.7)	52.9 (49.3–56.9)	73.2 (67.5–79.5)	57.6 (51.2–64.9)	63.3 (59.5–67.3)	50.7 (46.4–55.4)

CI, confidence interval; COVID-19, coronavirus disease 2019. Prevalence estimates are reported as total reports/total respondents (%), crude rates per 100 hospital discharges [estimate (95% CI)] and age-standardized rates per 100 hospital discharges [estimate (95% CI)].

At 12 months, dysosmia and dysgeusia were resolved in almost all patients though fatigue, altered consciousness and myalgia remained unresolved in more than 10% of the cohort. Males had greater recovery of symptoms at 12 months compared with females (Supplementary Table 5). The median time to the resolution of one or more neurological symptoms reported at acute hospitalization was generally longer in females than in males. Median times to symptom resolution generally increased with age, being the longest times reported by participants aged between 45 and 65 years (Fig. 2; Supplementary Fig. 6). Patients with more than one neurological symptom at hospitalization for acute COVID-19 had a prolonged time to resolution [5.2 months in females and 3.4 months in males; hazard ratio (HR) = 1.45; 95% CI = 1.31–1.60; Supplementary Table 5]. Patients who experienced neurological complications during hospital admission had a similar median time to recover compared with those who did not (Supplementary Fig. 7).

The cumulative probability of the resolution of altered consciousness was similar between females and males (Fig. 2; Supplementary Fig. 8). For all other symptoms, males had a far greater chance of symptom resolution at any given time.

### Multivariable analysis of PANSC

**Figure 3:**
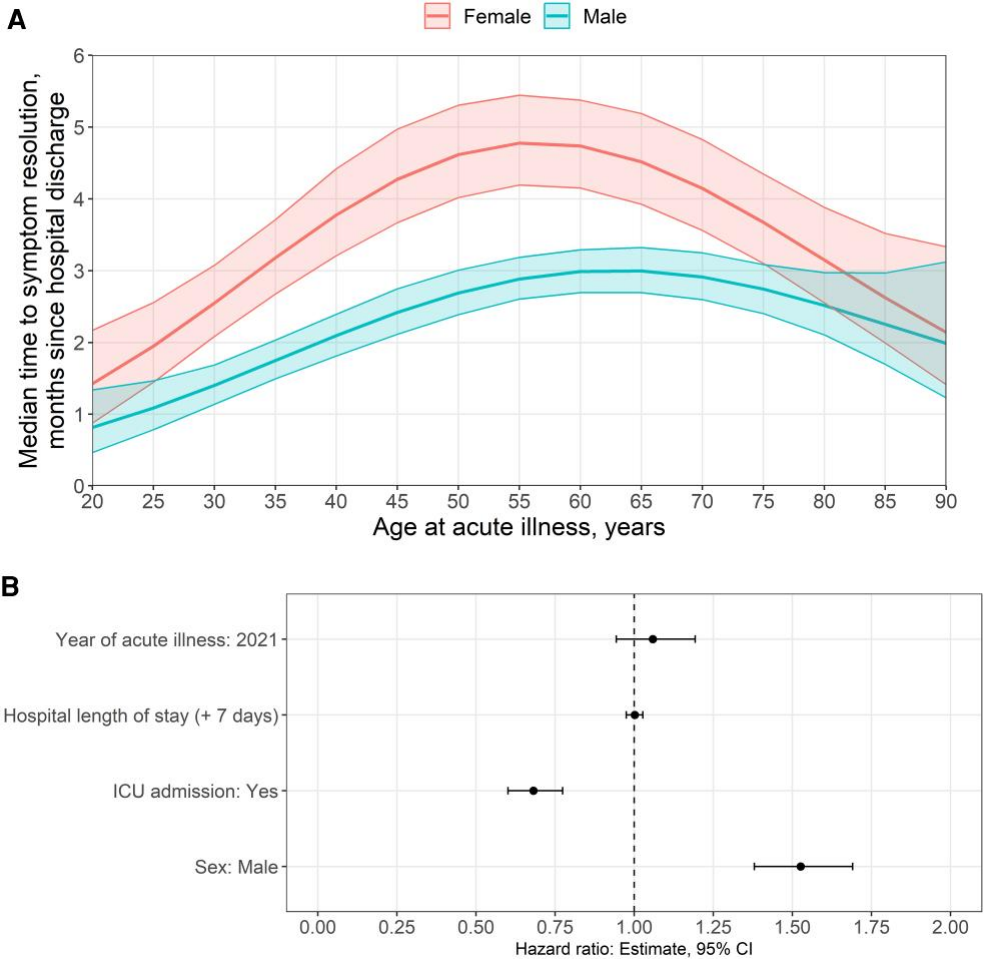
**Risk factors for time to symptom resolution.** (**A**) The median time (months, *y*-axis) to symptom resolution is based on the patient’s age (*x*-axis). (**B**) Multivariable analysis for time to symptom resolution for those with one or more neurological long COVID symptoms.

Male sex was significantly associated with a shorter time to the resolution of neurological symptoms (HR = 1.53; 95% CI = 1.39–1.69; Fig. 3; Supplementary Table 6). ICU admission and COVID-19 infection in 2021 (versus 2020), but not prolonged hospital length of stay, were significantly associated with a longer time to symptom resolution (Fig. 3). Multivariable results were similar between the full analysis cohort and the subset of participants evaluated for in-hospital neurological complications. The presence of in-hospital neurological complications was not associated with resolution time (HR = 1.16; 95% CI = 0.48–2.45; Supplementary Tables 6 and 7).

## Discussion

In this study, we present results from a large international multi-centre observational cohort of patients (ISARIC) who reported PANSC symptoms at regular time points over a 1-year period following an index hospitalization with acute COVID-19 disease. Our study is unique in reporting focused ‘neurological’ long COVID symptoms for up to 1 year and the analyses on time to symptom resolution. We showed that a large proportion of patients (70%) had more than one neurological symptom upon initial follow-up at ∼3 months, with females having higher frequencies and a longer time to the resolution (5.2 versus 3.4 months) of PANSC symptoms. The cumulative probability of the resolution of PANSC was far greater in males at any given time. This is in keeping with prior work where the female sex has been reported as a significant risk factor for long COVID (not specific to PANSC), with a meta-analysis of 7 studies and 386 234 patients demonstrating an odds ratio of 1.48 (95% CI = 1.17–1.86).^12,17^ Similarly, over half (1100 of 2103, 52%) of responders in a Latin American study reported that long COVID symptoms were more commonly reported by females (64.0%).^18^ The findings may be associated with females generally having more depression and anxiety; however, our study did not have those specific variables. The age group most affected with long COVID varied from young adults (21–40 years)^18^ to older adults (≥50 years)^19^ in different studies. It is noteworthy, unlike our study, that the aforementioned studies have not investigated specific PANSC, which may have resulted in some inconsistent findings on age. Our study demonstrated middle-aged participants (40–65 years) were more affected by PANSC in our study (Fig. 3).

Severe acute respiratory distress syndrome (ARDS) is a known risk factor for long-term cognitive and psychiatric impairment.^20,21^ Interestingly, we noted there were substantially more males than females who were admitted to ICU (31 versus 20%) and received mechanical ventilation (17 versus 12%; Table 1). This did not translate into more PANSC on follow-up, further highlighting the sex discrepancy in PANSC. Also, our study highlights that, in addition to a greater prevalence, symptom resolution takes longer in females than in males, which provides a new unique perspective to the current knowledge of long COVID, which was not reported in COVID-19 or general ARDS literature.

The aetiology of long COVID and of neurological symptoms of long COVID are areas of active research and are suspected to be multi-factorial.^22^ Several hypotheses implicate both the nervous system and the systemic pathogenic mechanisms such as SARS-CoV-2 viral persistence and neuroinvasion, abnormal immunological response (subacute to chronic), autoimmunity, coagulopathies and endotheliopathy with microvascular clot formation, which can contribute to hypoxic neuronal injury and blood–brain barrier dysfunction.^22^

Lastly, we identified that most survey participants were enrolled from high-income countries represented in the global ISARIC cohort. This geographical distribution differed from the total number of patients included in the WHO-ISARIC Clinical Characterization Protocol evaluated for neurological symptoms and in-hospital complications. Fewer survey participants from low-income and middle-income countries highlight the need to strengthen research capacity and develop strategies and tools to support effective patient follow-up in low-resource healthcare systems.^13^

### Limitations

This study has several limitations that should be considered when interpreting the main findings. A key among these is the methodological challenges of assessing patient symptoms and complications using remote clinician lead and self-assessments during pandemic constraints and common issues encountered when conducting multi-site longitudinal studies. First, as participant recruitment and follow-up were contingent on resourcing at individual study sites, data were collected at irregular intervals and subject to loss to follow-up. Second, it is likely that the study cohort was subject to a degree of selection bias, related to challenges in recruiting and retaining participants following acute COVID-19 hospitalization. We further considered population differences by reporting sex-specific prevalence as crude and age-standardized rates. Third, our study did not provide any mechanistic insights into long-term PANSC. Fourth, the rapid evolution of COVID-19 disease and its treatment with antiviral therapy such as nirmatrelvir or steroids were not well captured in the database, which limited our ability to investigate treatment impact on PANSC other than possible associations. Finally, multivariable analyses were limited in terms of adjustment for patient-level characteristics related to disease severity, for example, the use of mechanical ventilation. Limiting these analyses was intended to reduce the risk of reporting distorted associations from collider bias,^23^ common in observational studies.

Other study limitations relate to the scope of analyses possible on standardized data collections. First, the spectrum of neurological psychiatric manifestations and complications of COVID-19 is broader than the CRF terms included in the patient registry and survey. Secondly, due to the data collection methods used, mortality was recorded only if data collectors were notified of deaths before or while attempting to follow up with a participant. In cases where participants could not be contacted, the reason for loss to follow-up could not be determined from available data. Thirdly, it was not possible to assess the implications of PANSC on quality of life, as formal quality-of-life questionnaires and neurocognitive or psychiatric assessments suffered from significant missingness. Challenges exist in defining and capturing PANSC and establishing causation, especially in ICU patients with ARDS, where there is evidence of the long-term burden suffered by survivors. A more intense follow-up with defined endpoints would have allowed a more detailed analysis, but our study was a pragmatically driven extension of the initial WHO-ISARIC Clinical Characterization protocol (https://isaric.org/research/covid-19-clinical-research-resources/clinical-characterisation-protocol-ccp/).^24^ Most follow-up participants were from high-income countries, whereas the majority of patients in the ISARIC COVID registry were from low-income/middle-income countries. Finally, patient recruitment strategies varied between sites and were subject to staff and resource limitations, introducing the possibility of recruitment bias. As the heterogeneity of PANSC reported in the literature as it relates to long COVID is a challenge, our study with a large sample size (407 sites; 15 countries) offers an advantage. In a scoping review conducted in January 2021, out of 120 papers, there was 1 randomized clinical trial, 22 were cohort and 28 were cross-sectional studies, none of which focused on PANSC symptoms, thus highlighting the importance of our study.^25^

## Conclusion

This international multi-centre prospective cohort study demonstrates that PANSC after hospitalization is high, with a higher prevalence in females and a longer time to resolution. This supports the fact that females are disproportionately affected by PANSC, although males were admitted to ICU more often and were treated with mechanical ventilation more often than females. Stroke is an uncommon neurological complication after COVID-19 discharge. Given the burden of disease, further work is needed regarding the screening and treatment of long-term neurological symptoms after COVID, particularly in low-resource healthcare settings.

## Supplementary Material

fcae036_Supplementary_Data

## Data Availability

The data that underpin this analysis are highly detailed clinical data on individuals hospitalized with COVID-19. Due to the sensitive nature of these data and the associated privacy concerns, they are available via a governed data access mechanism following the review of a data access committee. Data can be requested via the IDDO COVID-19 Data Sharing Platform (http://www.iddo.org/covid-19). The Data Access Application, Terms of Access and details of the Data Access Committee are available on the website. Briefly, the requirements for access are a request from a qualified researcher working with a legal entity who has a health and/or research remit, and a scientifically valid reason for data access that adheres to appropriate ethical principles. The full terms can be accessed at https://www.iddo.org/document/covid-19-data-access-guidelines. A small subset of sites that contributed data to this analysis have not agreed to pooled data sharing as above. In the case of requiring access to these data, please contact the corresponding author in the first instance who will look to facilitate access.

## Appendix I

The members of the ISARIC Clinical Characterization Group are as follows: Laurent Abel, Amal Abrous, Kamal Abu Jabal, Hiba Abu Zayyad, Younes Ait Tamlihat, Aliya Mohammed Alameen, Marta Alessi, Beatrice Alex, Kévin Alexandre, Adam Ali, Kazali Enagnon Alidjnou, Clotilde Allavena, Nathalie Allou, Claire Andréjak, Andrea Angheben, François Angoulvant, Séverine Ansart, Jean-Benoît Arlet, Elise Artaud-Macari, Jean Baptiste Assie, Johann Auchabie, Hugues Aumaitre, Adrien Auvet, Eyvind W. Axelsen, Laurène Azemar, Cecile Azoulay, Benjamin Bach, Delphine Bachelet, Claudine Badr, Roar Bævre-Jensen, John Kenneth Baillie, Firouzé Bani-Sadr, Wendy S. Barclay, Marie Bartoli, Joaquín Baruch, Romain Basmaci, Jules Bauer, Alexandra Bedossa, Husna Begum, Sylvie Behilill, Anna Beltrame, Marine Beluze, Nicolas Benech, Delphine Bergeaud, José Luis Bernal Sobrino, Giulia Bertoli, Simon Bessis, Sybille Bevilcaqua, Karine Bezulier, Krishna Bhavsar, Zeno Bisoffi, Laurent Bitker, Mathieu Blot, Laetitia Bodenes, Debby Bogaert, Anne-Hélène Boivin, Isabela Bolaños, Pierre-Adrien Bolze, François Bompart, Raphaël Borie, Elisabeth Botelho-Nevers, Lila Bouadma, Olivier Bouchaud, Sabelline Bouchez, Damien Bouhour, Kévin Bouiller, Laurence Bouillet, Camile Bouisse, Anne-Sophie Boureau, Maude Bouscambert, Aurore Bousquet, Marielle Boyer-Besseyre, Axelle Braconnier, Sonja Hjellegjerde Brunvoll, Marielle Buisson, Danilo Buonsenso, Aidan Burrell, Ingrid G. Bustos, André Cabie, Eder Caceres, Cyril Cadoz, Jose Andres Calvache, Valentine Campana, Pauline Caraux-Paz, Nicolas Carlier, Thierry Carmoi, Marie-Christine Carret, Gail Carson, Maire-Laure Casanova, Guylaine Castor-Alexandre, François-Xavier Catherine, Paolo Cattaneo, Minerva Cervantes-Gonzalez, Anissa Chair, Catherine Chakveatze, Meera Chand, Jean-Marc Chapplain, Charlotte Charpentier, Julie Chas, Léo Chenard, Antoine Cheret, Thibault Chiarabini, Catherine Chirouze, Bernard Cholley, Marie-Charlotte Chopin, Yock Ping Chow, Barbara Wanjiru Citarella, Sara Clohisey, Gwenhaël Colin, Marie Connor, Anne Conrad, Graham S. Cooke, Hugues Cordel, Andrea Cortegiani, Grégory Corvaisier, Camille Couffignal, Sandrine Couffin-Cadiergues, Roxane Courtois, Stéphanie Cousse, Juan Luis Cruz Bermúdez, Jaime Cruz Rojo, Elodie Curlier, Ana da Silva Filipe, Charlene Da Silveira, Andrew Dagens, John Arne Dahl, Jo Dalton, Etienne De Montmollin, Cristina De Rose, Thushan de Silva, Alexa Debard, Marie-Pierre Debray, Nathalie DeCastro, Romain Decours, Eve Defous, Isabelle Delacroix, Eric Delaveuve, Karen Delavigne, Christelle Delmas, Pierre Delobel, Elisa Demonchy, Emmanuelle Denis, Dominique Deplanque, Diane Descamps, Mathilde Desvallées, Alpha Diallo, Sylvain Diamantis, Fernanda Dias Da Silva, Kévin Didier, Jean-Luc Diehl, Jérôme Dimet, Vincent Dinot, Fara Diop, Alphonsine Diouf, Félix Djossou, Annemarie B. Docherty, Christl A. Donnelly, Céline Dorival, Eric D’Ortenzio, Nathalie Dournon, Thomas Drake, Amiel A. Dror, Vincent Dubee, François Dubos, Alexandre Ducancelle, Susanne Dudman, Paul Dunand, Jake Dunning, Bertrand Dussol, Xavier Duval, Anne Margarita Dyrhol-Riise, Michael Edelstein, Linn Margrete Eggesbø, Mohammed El Sanharawi, Brigitte Elharrar, Merete Ellingjord-Dale, Philippine Eloy, Isabelle Enderle, Ilka Engelmann, Vincent Enouf, Olivier Epaulard, Hélène Esperou, Marina Esposito-Farese, Manuel Etienne, Mirjam Evers, Marc Fabre, Isabelle Fabre, Cameron J. Fairfield, Karine Faure, Raphaël Favory, François-Xavier Ferrand, Eglantine Ferrand Devouge, Nicolas Ferriere, Céline Ficko, William Finlayson, Thomas Flament, Tom Fletcher, Aline-Marie Florence, Erwan Fourn, Robert A. Fowler, Christophe Fraser, Stéphanie Fry, Valérie Gaborieau, Rostane Gaci, Jean-Charles Gagnard, Amandine Gagneux-Brunon, Sérgio Gaião, Linda Gail Skeie, Carrol Gamble, Noelia García Barrio, Esteban Garcia-Gallo, Denis Garot, Valérie Garrait, Anatoliy Gavrylov, Alexandre Gaymard, Eva Geraud, Louis Gerbaud Morlaes, Jade Ghosn, Tristan Gigante, Guillermo Giordano, Michelle Girvan, Valérie Gissot, Daniel Glikman, François Goehringer, Kyle Gomez, Marie Gominet, Yanay Gorelik, Isabelle Gorenne, Laure Goubert, Cécile Goujard, Tiphaine Goulenok, Pascal Granier, Christopher A. Green, William Greenhalf, Segolène Greffe, Fiona Griffiths, Jérémie Guedj, Martin Guego, Romain Guery, Anne Guillaumot, Laurent Guilleminault, Thomas Guimard, Ali Hachemi, Nadir Hadri, Matthew Hall, Sophie Halpin, Rebecca Hamidfar, Bato Hammarström, Hayley Hardwick, Ewen M. Harrison, Janet Harrison, Lars Heggelund, Ross Hendry, Maxime Hentzien, Diana Hernandez, Liv Hesstvedt, Rupert Higgins, Hikombo Hitoto, Antonia Ho, Alexandre Hoctin, Isabelle Hoffmann, Jan Cato Holter, Peter Horby, Ikram Houas, Jean-Sébastien Hulot, Samreen Ijaz, Patrick Imbert, Mariachiara Ippolito, Margaux Isnard, Mette Stausland Istre, Danielle Jaafar, Salma Jaafoura, Julien Jabot, Clare Jackson, Stéphane Jaureguiberry, Florence Jego, Synne Jenum, Silje Bakken Jørgensen, Cédric Joseph, Mercé Jourdain, Ouifiya Kafif, Florentia Kaguelidou, Sabina Kali, Deepjyoti Kalita, Karl Trygve Kalleberg, Christiana Kartsonaki, Seán Keating, Sadie Kelly, Kalynn Kennon, Younes Kerroumi, Antoine Khalil, Saye Khoo, Beathe Kiland Granerud, Anders Benjamin Kildal, Antoine Kimmoun, Eyrun Floerecke Kjetland Kjetland, Paul Klenerman, Gry Kloumann Bekken, Stephen R. Knight, Arsène Kpangon, Oksana Kruglova, Galyna Kutsyna, Marie Lachatre, Marie Lacoste, Nadhem Lafhej, Marie Lagrange, Fabrice Laine, Olivier Lairez, Antonio Lalueza, Marc Lambert, Marie Langelot-Richard, Vincent Langlois, Cédric Laouénan, Samira Laribi, Delphine Lariviere, Stéphane Lasry, Odile Launay, Didier Laureillard, Yoan Lavie-Badie, Andy Law, Minh Le, Clément Le Bihan, Cyril Le Bris, Georges Le Falher, Lucie Le Fevre, Quentin Le Hingrat, Marion Le Maréchal, Soizic Le Mestre, Gwenaël Le Moal, Vincent Le Moing, Hervé Le Nagard, Jennifer Lee, Gary Leeming, Laurent Lefebvre, Bénédicte Lefebvre, Benjamin Lefèvre, Sylvie LeGac, Jean-Daniel Lelievre, Adrien Lemaignen, Véronique Lemee, Anthony Lemeur, Marc Leone, Quentin Lepiller, François-Xavier Lescure, Olivier Lesens, Mathieu Lesouhaitier, Sophie Letrou, Yves Levy, Bruno Levy, Claire Levy-Marchal, Erwan L’Her, Geoffrey Liegeon, Wei Shen Lim, Bruno Lina, Andreas Lind, Guillaume Lingas, Sylvie Lion-Daolio, Marine Livrozet, Paul Loubet, Bouchra Loufti, Guillame Louis, Jean Christophe Lucet, Carlos Lumbreras Bermejo, Miles Lunn, Liem Luong, Dominique Luton, Moïse Machado, Gabriel Macheda, Guillermo Maestro de la Calle, Rafael Mahieu, Sophie Mahy, Mylène Maillet, Thomas Maitre, Denis Malvy, Victoria Manda, Laurent Mandelbrot, Julie Mankikian, Aldric Manuel, Samuel Markowicz, John Marshall, Guillaume Martin-Blondel, Martin Martinot, Olga Martynenko, Mathieu Mattei, Laurence Maulin, Thierry Mazzoni, Colin McArthur, Sarah E. McDonald, Kenneth A. McLean, Cécile Mear-Passard, France Mentré, Alexander J. Mentzer, Noémie Mercier, Emmanuelle Mercier, Antoine Merckx, Mayka Mergeay-Fabre, Laura Merson, Roberta Meta, Agnès Meybeck, Alison M. Meynert, Vanina Meysonnier, Mehdi Mezidi, Céline Michelanglei, Isabelle Michelet, Sarah Moore, Shona C. Moore, Lina Morales Cely, Lucia Moro, Hugo Mouquet, Clara Mouton Perrot, Julien Moyet, Jimmy Mullaert, Fredrik Müller, Karl Erik Müller, Marlène Murris, Srinivas Murthy, Nadège Neant, Anthony Nghi, Duc Nguyen, Alistair D. Nichol, Mahdad Noursadeghi, Saad Nseir, Elsa Nyamankolly, Anders Benteson Nygaard, Piero L. Olliaro, Wilna Oosthuyzen, Peter Openshaw, Claudia Milena Orozco-Chamorro, Paul Otiku, Nadia Ouamara, Rachida Ouissa, Eric Oziol, Maïder Pagadoy, Justine Pages, Massimo Palmarini, Prasan Kumar Panda, Nathalie Pansu, Aurélie Papadopoulos, Rachael Parke, Jérémie Pasquier, Bruno Pastene, Christelle Paul, William A. Paxton, Jean-François Payen, Miguel Pedrera Jiménez, Florent Peelman, Nathan Peiffer-Smadja, Vincent Peigne, Daniel Perez, Thomas Perpoint, Vincent Pestre, Ventzislava Petrov-Sanchez, Frank Olav Pettersen, Gilles Peytavin, Walter Picard, Olivier Picone, Lionel Piroth, Chiara Piubelli, Riinu Pius, Laurent Plantier, Julien Poissy, Ryadh Pokeerbux, Georgios Pollakis, Diane Ponscarme, Sébastien Preau, Mark G. Pritchard, Víctor Quirós González, Else Quist-Paulsen, Christian Rabaud, Marie Rafiq, Blandine Rammaert, Christophe Rapp, Stanislas Rebaudet, Sarah Redl, Dag Henrik Reikvam, Martine Remy, Anne-Sophie Resseguier, Matthieu Revest, Luis Felipe Reyes, Antonia Ricchiuto, Laurent Richier, Patrick Rispal, Karine Risso, Stephanie Roberts, David L. Robertson, Olivier Robineau, Paola Rodari, Pierre-Marie Roger, Amanda Rojek, Roberto Roncon-Albuquerque Jr, Mélanie Roriz, Manuel Rosa-Calatrava, Andrea Rossanese, Patrick Rossignol, Carine Roy, Benoît Roze, Clark D. Russell, Aleksander Rygh Holten, Charlotte Salmon Gandonniere, Hélène Salvator, Olivier Sanchez, Vanessa Sancho-Shimizu, Pierre-François Sandrine, Oana Sandulescu, Benjamine Sarton, Egle Saviciute, Arnaud Scherpereel, Marion Schneider, Janet T. Scott, James Scott-Brown, Nicholas Sedillot, Malcolm G. Semple, Eric Senneville, Pablo Serrano Balazote, Catherine A. Shaw, Victoria Shaw, Girish Sindhwani, Nassima Si Mohammed, Jeanne Sibiude, Louise Sigfrid, Dario Sinatti, Vegard Skogen, Sue Smith, Lene Bergendal Solberg, Tom Solomon, Agnès Sommet, Arne Søraas, Albert Sotto, Edouard Soum, Elisabetta Spinuzza, Shiranee Sriskandan, Sarah Stabler, Trude Steinsvik, Birgitte Stiksrud, Adrian Streinu-Cercel, Anca Streinu-Cercel, David Stuart, Richa Su, Charlotte Summers, Lysa Tagherset, Renaud Tamisier, Coralie Tardivon, Pierre Tattevin, Marie-Capucine Tellier, François Téoulé, Olivier Terrier, Nicolas Terzi, Vincent Thibault, Simon-Djamel Thiberville, Benoît Thill, Emma C. Thomson, Mathew Thorpe, Ryan S. Thwaites, Vadim Tieroshyn, Jean-François Timsit, Noémie Tissot, Kristian Tonby, Cécile Tromeur, Tiffany Trouillon, Jeanne Truong, Christelle Tual, Sarah Tubiana, Jean-Marie Turmel, Lance C. W. Turtle, Anders Tveita, Timothy M. Uyeki, Piero Valentini, Sylvie Van Der Werf, Noémie Vanel, Charline Vauchy, Aurélie Veislinger, Benoit Visseaux, Fanny Vuotto, Steve Webb, Jia Wei, Murray Wham, Paul Henri Wicky, Aurélie Wiedemann, Natalie Wright, Yazdan Yazdanpanah, Cécile Yelnik, Hodane Yonis, Marion Zabbe, Maria Zambon and David Zucman.
