# Supplementary material for: Sex differences in post-acute neurological sequelae of SARS-CoV-2 and symptom resolution in adults after coronavirus disease 2019 hospitalization: an international multi-centre prospective observational study
Source: Brain Commun. 2024 Feb 9;6(2):fcae036. doi: 10.1093/braincomms/fcae036 (PMC10914448; doi:10.1093/braincomms/fcae036)

**SUPPLEMENT: Sex Differences in Neurological Long COVID and Symptom Resolution in Adults after COVID-19 Hospitalization**

**SUPPLEMENTAL FILES:**

**Supplemental File 1.** Additional statistical methods

**SUPPLEMENTAL TABLES**

**Supplemental Table 1.** Data completeness summary.

**Supplemental Table** **2**. Total number of follow-up surveys per participant and the median days of follow-up in our study cohort.

**Supplemental Table 3.** Extended baseline characteristics for survey participants

**Supplemental Table 4**. In-hospital neurological complications and new cases of stroke reported during survey follow-up.

**Supplemental Table 5.** Time to symptom resolution (months since hospital. discharge for acute COVID-19) and frequency of resolution at 3-, 6- and 12-month intervals (stratified by sex).

**Supplemental Table 6**. Multivariable cox regression for time to symptom resolution

**Supplemental Table 7.** Observed prevalence and median time to symptom resolution stratified by reported in-hospital neurological complications.

**SUPPLEMENTAL FIGURES**

**Supplemental Figure 1.** Distribution of survey follow-up times.

**Supplemental Figure 2.** Timing of acute COVID-19 hospitalization for all survey participants evaluated for neurological symptoms and/or complications stratified by Sex (A) and ICU admission (B) at any time during hospitalization.

**Supplemental Figure 3.** Observed pairwise prevalence of neurological symptoms reported at hospital admission at the time of acute COVID-19.

**Supplemental Figure 4.** Reported prevalence (%) of other neurological symptoms at initial follow-up survey.

**Supplemental Figure 5. A.** Median times to symptom resolution by age and sex, in months since acute hospital discharge: primary analysis. Sample sizes by symptom are 373 (Altered consciousness/confusion), 1,208 (Dysgeusia), 1,079 (Anosmia), 2,228 (Myalgia), 3,375 (Fatigue), 4,410 (One or more neurological symptoms)**.**

Median times to symptom resolution by age and sex, in months since acute hospital discharge: primary analysis. **B.** Median times to symptom resolution by age and sex, in months since acute hospital discharge: sensitivity analysis. Sample sizes by symptom are 373 (Altered consciousness/confusion), 1,208 (Dysgeusia), 1,079 (Anosmia), 2,228 (Myalgia), 3,375 (Fatigue), 4,410 (One or more neurological symptoms)**.**

**Supplemental Figure 6.** Cumulative probabilities for time to symptom resolution for patients without (n = 896, left panel) and with neurological complications (meningitis/encephalitis, seizure or stroke) (n = 15; right panel) during hospital admission for acute COVID-19; primary analysis*. The left panel shows results for matched controls.

**Supplemental Figure 7.** Cumulative probabilities for time to symptom resolution, sensitivity analysis. Primary analysis is displayed in **Figure 2** (manuscript). Sample sizes by symptom are 373 (Altered consciousness/confusion), 1,208 (Dysgeusia), 1,079 (Anosmia), 2,228 (Myalgia), 3,375 (Fatigue), 4,410 (One or more neurological symptoms)

**Supplemental Figure 8.** Cumulative participant loss to survey follow-up, months since acute COVID-19 hospital discharge (n=6,862). The probability of participant loss increases over time from the discharge.

**Supplemental File 1: Additional statistical methods**

*Prevalence estimates*

Crude and age-standardised prevalence rates were estimated per 100 hospital discharges as the common denominator. Estimates and 95% confidence intervals assumed rates were Gamma distributed.

Age standardization required knowledge of age-sex distributions of the COVID-19 hospitalized population. Population weights for a given study group were estimated from all surveyed and non-surveyed individuals with data collected by the ISARIC-WHO Clinical Characterization Protocol, subject to the same inclusion/exclusion hospitalization criteria. Weights were computed for the age bands 18-30, 31-40, 41-50, 51-60, 61-70, 70-80, and 80+. Confidence intervals for age-adjusted rates were computed by the Fay and Feuer method.

*Time to symptom resolution*

Analysis of symptom resolution considered participants with neurological symptoms reported at initial acute hospitalization only (n = 3,542; **Table 2**). Analysis considered times to symptom resolution per neurological symptom, and for one or more neurological symptoms evaluated at hospitalization as a composite measure. Times were modelled in months since hospital discharge.

The survey study design meant that the exact times of symptom resolution were unknown. For participants who reported symptom resolution, we assumed that times to symptom resolution occurred before the initial follow-up survey was completed or fell between consecutive survey time points. Based on these data, the following forms of censoring were applied:

1. Participants who reported symptom resolution before the time of the initial follow-up survey were left censored at hospital discharge.
2. Participants who reported symptom resolution between consecutive surveys was interval censored. Interval bounds were defined by consecutive survey times where a symptom report changed from present to absent.
3. Participants who reported a symptom being present at their last known survey time were right-censored, or at 12 months after hospital discharge, whichever occured first.

Data were modelled by Weibull parametric regression, with sex as a fixed effect. Analysis was completed using the icenReg R package, which offers parametric and semi-parametric approaches to survival regression for interval-censored data. Model results were summarised by cumulative probability functions up to 12 months after hospital discharge. Reported median times and corresponding 95% confidence intervals were extracted from model fits.

We considered model sensitivity based on two definitions of symptom resolution:

1. Primary analysis: symptom resolution was defined by the first survey date where a participant reported the symptom as absent;
2. Sensitivity analysis: symptom resolution was defined by participant responses at their last known date of survey follow-up.

An example of the difference between these two definitions is shown in the schematic below. The definition of symptom resolution under our sensitivity analysis implied longer times to symptom resolution.


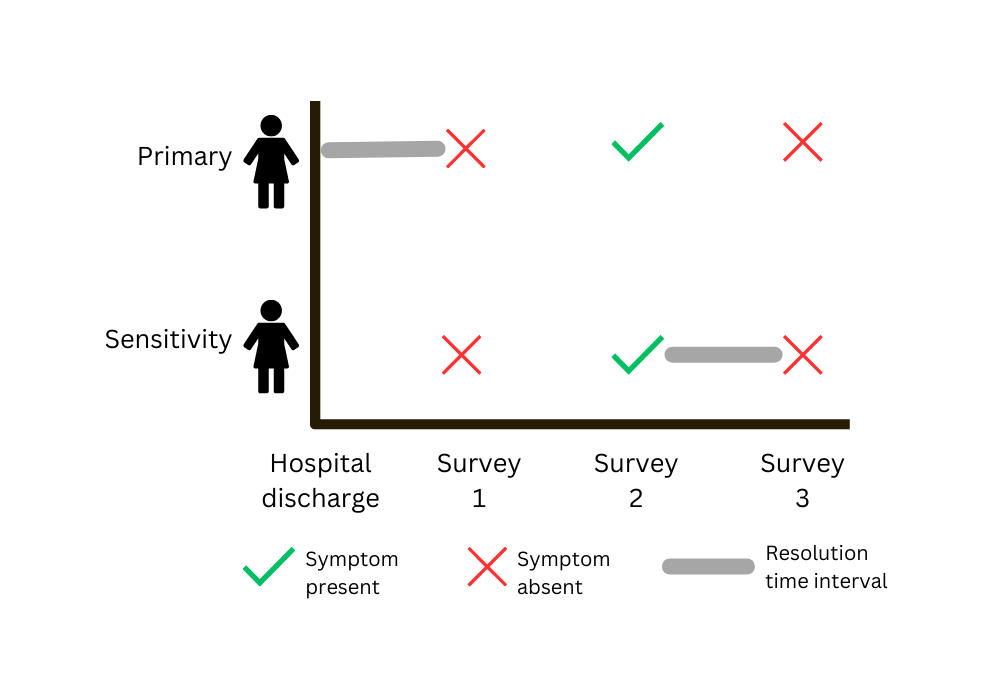


Differences in definition affected between 3 and 11% of included participants depending on the symptom(s) analysed.

*Matched case-controls analysis for in-hospital neurological complications*

We analysed times to symptom resolution among survey participants who were evaluated for neurological complications during acute COVID-19 hospitalization. Outcomes for participants who reported one or more in-hospital neurological complications were compared with a matched group of participants who did not report any in-hospital complications. Participants were matched on sex, calendar period of hospitalization (6 monthly intervals), age at hospitalization (5-year age bands), and the presence of one or more neurological symptoms at hospitalization.

**Supplemental Table 1:** **Data completeness summary**

Data availability was assessed for cohort characteristics collected at initial acute hospitalization for COVID-19 illness. Data availability on each characteristic was summarised by the total number of participants with the characteristic recorded in the data (e.g., Age in years, ECMO: ‘Yes’ or ‘No’). Corresponding percentages for each characteristic represents variable completeness in the analysis cohort defined in **Figure 1** and **Table 1**.

| Characteristic | Full cohort  (n =6,862) | Female  (n = 2,691) | Male  (n = 4,171) |
| --- | --- | --- | --- |
| **Demographics** | | | |
| Age, years | 6,862  (100%) | 2,691 (100%) | 4,171 (100%) |
| High-income country | 6,862  (100%) | 2,691 (100%) | 4,171 (100%) |
| Asia | 1,652 (100%) | 573  (100%) | 1,077 (100%) |
| Europe | 5,092 (100%) | 2,055 (100%) | 3,037 (100%) |
| Africa | 77 (100%) | 46 (100%) | 31 (100%) |
| Latin America/Caribbean | 43 (100%) | 17 (100%) | 26 (100%) |
| **Comorbidities** | | | |
| Asthma | 6,837  (99.6%) | 2,682 (99.7%) | 4,155 (99.6%) |
| Chronic cardiac disease^a^ | 6,837  (99.6%) | 2,681 (99.6%) | 4,156 (99.6%) |
| Chronic kidney disease^b^ | 6,848  (99.8%) | 2,683 (99.7%) | 4,165 (99.9%) |
| Chronic neurological disorder^c^ | 6,836  (99.6%) | 2,681 (99.6%) | 4,155 (99.6%) |
| Chronic pulmonary disease^d^ | 6,848  (99.8%) | 2,684 (99.7%) | 4,164 (99.8%) |
| Dementia | 6,844  (99.7%) | 2,682 (99.7%) | 4,162 (99.8%) |
| Diabetes mellitus | 4,546  (66.2%) | 1,740 (64.7%) | 2,806 (67.3%) |
| Hypertension | 6,814  (99.3%) | 2,673 (99.3%) | 4,141 (99.3%) |
| Liver disease^e^ | 6,763  (98.6%) | 2,644 (98.3%) | 4,119 (98.8%) |
| Malignant Neoplasm^f^ | 6,838  (99.7%) | 2,681 (99.6%) | 4,157 (99.7%) |
| Obesity | 2,701  (39.4%) | 1,088 (40.4%) | 1,613 (38.7%) |
| Smoking^g^ | 2,389  (34.8%) | 982  (36.5%) | 1,407 (33.7%) |
| **Neurological symptoms at hospital admission** | | | |
| Altered consciousness | 6,608 (96.3%) | 2,596 (96.5%) | 4,013 (96.2%) |
| Fatigue | 6,572 (95.8%) | 2,586 (96.1%) | 3,986 (95.6%) |
| Anosmia | 6,071  (88.5%) | 2,387 (88.7%) | 3,684 (88.3%) |
| Dysgeusia | 6,073  (88.5%) | 2,393 (88.9%) | 3,680 (88.2%) |
| Myalgia | 6,479 (99.4%) | 2,562 (95.2%) | 3,917 (93.9%) |
| Seizure | 4,981 (72.6%) | 1,998 (74.2%) | 2,983 (71.5%) |
| 1 or more neurological symptoms | 6,862 (100%) | 2,691 (100%) | 4,171 (100%) |
| **Neurological complications during hospitalization** | | | |
| CNS infection | 2,516  (36.7%) | 1,047 (38.9%) | 1,469  (35.2%) |
| Stroke | 2,501 (36.4%) | 1,044  (38.8%) | 1,457  (34.9%) |
| Seizure | 2,516  (36.7%) | 1,046 (38.9%) | 1,470  (35.2%) |
| 1 or more neurological complications | 2,520 (36.7%) | 1,049  (39.0%) | 1,471 (35.3%) |
| **COVID-19 index hospitalization information** | | | |
| ICU admission | 6,862 (100%) | 2,691 (100%) | 4,171 (100%) |
| Mechanically ventilated | 6,572 (95.8%) | 2,558 (95.1%) | 4,014 (96.2%) |
| ECMO | 1,053 (15.3%) | 426  (15.8%) | 627  (15.0%) |
| Hospital length of stay, days | 6,862 (100%) | 2,691 (100%) | 4,171 (100%) |
| Antiviral use | 5,121 (74.6%) | 2,005 (74.5%) | 3,116 (74.7%) |
| Corticosteroid use | 4,092 (59.6%) | 1,607 (59.7%) | 2,485 (59.6%) |

ICU=Intensive Care Unit, ECMO=Extracorporeal membrane oxygenation

^a^ Chronic cardiac disease: any of coronary artery disease, heart failure, congenital heart disease, cardiomyopathy, or rheumatic heart disease (not hypertension)

^b^ Chronic kidney disease: chronic estimated glomerular filtration rate < 60mL/min/1.73 m^2^ or history of kidney transplantation.

^c^ Chronic neurologic disorder: any of cerebral palsy, multiple sclerosis, motor neuron disease, muscular dystrophy, myasthenia gravis, Parkinson’s disease, stroke, severe learning difficulty.

^d^ Chronic pulmonary disease (not asthma): any of chronic obstructive pulmonary disease (chronic bronchitis, chronic obstructive pulmonary disease (COPD), emphysema), cystic fibrosis, bronchiectasis, interstitial lung disease, pre‐existing requirement for long term oxygen therapy. Not including asthma.

^e^ Cirrhosis without portal hypertension or chronic hepatitis.

^f^ Current solid organ or haematological malignancy. Please do not include malignancies that have been declared ‘cured’ ≥5 years ago with no evidence of ongoing disease. Does not included non‐melanoma skin cancers. Does not include benign growths or dysplasia.

^g^ At least one cigarette, cigar, pipe or equivalent per day before the onset of the current illness.

^h^ Subset of respondents evaluated for in-hospital complications (n = 2,520; **Figure 1**).

**Supplemental Table 2.** Total number of follow-up surveys per participant and the median days of follow-up in our study cohort. Times are summarised as medians with lower and upper quartiles.

| Survey number | Total respondents | Time since COVID-19 hospital discharge, days |
| --- | --- | --- |
| 1 | 6,862 | 102 (77.0 to 183.0) |
| 2 | 3,344 | 239 (175.0 to 388.0) |
| 3 | 387 | 371 (295.0 to 417.5) |
| 4 | 50 | 367 (362.2 to 377.8) |

COVID-19: Coronavirus Disease 2019

**Supplemental Table 3.** Extended Baseline characteristics for survey participants.

| Characteristic | Full cohort | Female | Male |
| --- | --- | --- | --- |
| Total survey participants | 6,862 | 2,691 | 4,171 |
| Geographic sub-region, n (%) | | | |
| South-eastern Asia | 30 (0.4) | 13 (0.5) | 17 (0.4) |
| Southern Asia | 1,422 (20.7) | 460 (17.1) | 962 (23.1) |
| Western Asia | 198 (2.9) | 100 (3.7) | 98 (2.3) |
| Eastern Europe | 122 (1.8) | 77 (2.9) | 45 (1.1) |
| Northern Europe | 2,970 (43.3) | 1,182 (43.9) | 1,788 (42.9) |
| Southern Europe | 248 (3.6) | 99 (3.7) | 149 (3.6) |
| Western Europe | 1,752 (25.5) | 697 (25.9) | 1,055 (25.3) |
| Northern Africa | 3 (0.0) | 2 (0.1) | 1 (0.0) |
| Sub-Saharan Africa | 74 (1.1) | 44 (1.6) | 30 (0.7) |
| Latin America and the Caribbean | 43 (0.6) | 17 (0.6) | 26 (0.6) |
| Comorbidities, n (%) | | | |
| Asthma | 759 (11.1) | 411 (15.3) | 348 (8.4) |
| Chronic pulmonary disease ^a^ | 503 (7.3) | 153 (5.7) | 350 (8.4) |
| Dementia | 20 (0.3) | 15 (0.6) | 5 (0.1) |
| Hypertension | 2,187 (32.1) | 770 (28.8) | 1,417 (34.2) |
| Liver disease ^b^ | 107 (1.6) | 44 (1.7) | 63 (1.5) |
| Malignant neoplasm ^c^ | 195 (2.9) | 74 (2.8) | 121 (2.9) |
| Smoking ^d^ | 671 (28.1) | 161 (16.4) | 510 (36.2) |

^a^ Chronic pulmonary disease (not asthma): any of chronic obstructive pulmonary disease (chronic bronchitis, chronic obstructive pulmonary disease (COPD), emphysema), cystic fibrosis, bronchiectasis, interstitial lung disease, pre‐existing requirement for long term oxygen therapy. Not including asthma.

^b^ Cirrhosis without portal hypertension or chronic hepatitis.

^c^ Current solid organ or haematological malignancy. Please do not include malignancies that have been declared ‘cured’ ≥5 years ago with no evidence of ongoing disease. Does not included non‐melanoma skin cancers. Does not include benign growths or dysplasia.

^d^ At least one cigarette, cigar, pipe or equivalent per day before the onset of the current illness.

**Supplemental Table 4**. In-hospital neurological complications and new cases of stroke reported during survey follow-up.

| Characteristic | All participants | Female | Male |
| --- | --- | --- | --- |
| **Reported during acute COVID-19 hospitalization** | | | |
| Total participants evaluated | 2,520 | 1,049 | 1,471 |
| 1+ neurological complications ^a^ | 27/2,520  1.07 (0.74 to 1.56) | 11/1,049  1.05 (0.58 to 1.88) | 16/1,471  1.09 (0.67 to 1.77) |
| Meningitis/encephalitis | 8/2,516  0.32 (0.16 to 0.63) | 3/1,047  0.29 (0.09 to 0.88) | 5/1,469  0.34 (0.14 to 0.82) |
| Stroke | 12/2,516  0.48 (0.27 to 0.84) | 3/1,046  0.29 (0.09 to 0.89) | 9/1,470  0.61 (0.32 to 1.17) |
| Seizures | 9/2,501  0.36 (0.19 to 0.69) | 6/1,044  0.57 (0.26 to 1.27) | 3/1,457  0.21 (0.07 to 0.64) |
| **Reported during survey follow-up** | | | |
| Deep vein thrombosis | 21/2,383 (0.9%) | 5/910 (0.5%) | 16/1,473 (1.1%) |
| Heart attack | 16/2,388 (0.7%) | 3/912 (0.3%) | 13/1,476 (0.9%) |
| Pulmonary embolism | 36/2,386 (1.5%) | 12/909 (1.3%) | 24/1,477 (1.6%) |
| Kidney problems | 45/2,382 (1.9%) | 16/906 (1.8%) | 29/1,476 (2.0%) |
| Stroke/Transient Ischemic Attack | 11/2,390 (0.5%) | 3/912 (0.3%) | 8/1,478 (0.5%) |

^a^ Meningitis/encephalitis, in-hospital seizures, or stroke

**Supplemental Table 5.** Median times to symptom resolution (months since hospital discharge for acute COVID-19) and percentage of cohort with symptom resolution at 3-, 6- and 12-months (stratified by sex). Time to resolution is adjusted for age at hospital admission (60 years). Results presented are model-based estimates from a Weibull regression model assuming mixed-case censoring: median times correspond to the 50th percentile of the parametric survival curve (Estimate, 95% CI). Symptom resolution percentages represent fitted survival estimates at 3, 6 and 12 months (Estimate, 95% CI). Participants analysed correspond to the number of participants with a neurological symptom present at initial hospitalization or at initial follow-up survey within 30 days of acute COVID-19 hospital discharge, if symptom information at initial hospitalization was missing (see “Methods: Statistical Analysis”; n = 1,785 (Female), 2,625 (Male).

| Neurological symptom^a^ | Female | | Male | |
| --- | --- | --- | --- | --- |
| Symptom resolution definition^b^ | Primary | Sensitivity | Primary | Sensitivity |
| Altered consciousness/confusion | | | | |
| Participants analysed | 144 | 144 | 229 | 229 |
| Median, months (95% CI) | 3.9 (2.8 to 5.2) | 5.3 (3.9 to 7.0) | 3.4 (2.6 to 4.4) | 4.7 (3.7 to 6.0) |
| 3 months (%) | 43 (33 to 53) | 33 (25 to 42) | 46 (38 to 54) | 36 (28 to 44) |
| 6 months (%) | 65 (55 to 76) | 55 (45 to 66) | 69 (60 to 77) | 59 (50 to 68) |
| 12 months (%) | 86 (77 to 94) | 80 (69 to 90) | 89 (82 to 95) | 83 (75 to 91) |
| Fatigue | | | | |
| Participants analysed | 1,372 | 1,372 | 2,003 | 2,003 |
| Median, months (95% CI) | 3.4 (3.1 to 3.7) | 4.7 (4.3 to 5.2) | 2.4 (2.2 to 2.6) | 3.4 (3.1 to 3.6) |
| 3 months (%) | 46 (43 to 49) | 35 (32 to 38) | 58 (55 to 60) | 46 (43 to 49) |
| 6 months (%) | 71 (67 to 74) | 59 (55 to 62) | 82 (80 to 84) | 72 (69 to 74) |
| 12 months (%) | 91 (89 to 94) | 84 (81 to 87) | 97 (96 to 98) | 93 (91 to 94) |
| Anosmia | | | | |
| Participants analysed | 450 | 450 | 629 | 629 |
| Median, months (95% CI) | 1.4 (1.2 to 1.7) | 1.6 (1.3 to 1.9) | 1.0 (0.8 to 1.2) | 1.2 (1.0 to 1.4) |
| 3 months (%) | 76 (70 to 81) | 72 (66 to 77) | 87 (84 to 91) | 82 (78 to 86) |
| 6 months (%) | 94 (91 to 96) | 92 (88 to 95) | 98 (97 to 99) | 96 (95 to 98) |
| 12 months (%) | 100 (99 to 100) | 99 (98 to 100) | 100 (100 to 100) | 100 (100 to 100) |
| Dysgeusia | | | | |
| Participants analysed | 506 | 506 | 702 | 702 |
| Median, months (95% CI) | 1.2 (1.0 to 1.5) | 1.5 (1.2 to 1.7) | 1.1 (0.9 to 1.2) | 1.2 (1.0 to 1.4) |
| 3 months (%) | 81 (76 to 85) | 74 (69 to 79) | 86 (82 to 89) | 81 (77 to 85) |
| 6 months (%) | 96 (94 to 98) | 93 (90 to 95) | 98 (97 to 99) | 96 (94 to 97) |
| 12 months (%) | 100 (100 to 100) | 99 (99 to 100) | 100 (100 to 100) | 100 (100 to 100) |
| Myalgia | | | | |
| Participants analysed | 978 | 978 | 1,250 | 1,250 |
| Median, months (95% CI) | 2.9 (2.5 to 3.3) | 4.2 (3.7 to 4.8) | 2.2 (2.0 to 2.5) | 2.9 (2.6 to 3.3) |
| 3 months (%) | 51 (48 to 55) | 41 (37 to 44) | 59 (56 to 62) | 51 (48 to 54) |
| 6 months (%) | 72 (68 to 76) | 61 (57 to 65) | 80 (77 to 82) | 72 (68 to 75) |
| 12 months (%) | 89 (87 to 92) | 81 (77 to 85) | 94 (92 to 96) | 89 (87 to 92) |
| One or more neurological symptoms | | | | |
| Participants analysed | 1,785 | 1,785 | 2,625 | 2,625 |
| Median, months (95% CI) | 5.2 (4.7 to 5.7) | 6.7 (6.0 to 7.4) | 3.4 (3.2 to 3.7) | 4.6 (4.3 to 5.0) |
| 3 months (%) | 35 (32 to 37) | 28 (25 to 30) | 46 (44 to 48) | 37 (35 to 39) |
| 6 months (%) | 55 (52 to 58) | 47 (43 to 50) | 68 (66 to 71) | 59 (56 to 61) |
| 12 months (%) | 77 (74 to 81) | 70 (66 to 74) | 88 (86 to 90) | 82 (79 to 85) |

CI: Confidence interval

^a^ Definitions for Neurological symptoms at hospital admission are provided in **Supplemental File 1**.

^b^ See **Supplemental File 2** for the rationale behind primary and sensitivity analyses.

**Supplemental Table 6**: Multivariable cox regression for time to symptom resolution; one or more neurological symptoms evaluated at hospitalization. Age-related trends are presented in **Figure 3.**

| Parameter | All respondents with neurological symptoms present at hospitalization  HR (95% CI) | Respondents with neurological symptoms present at hospitalization, data available on in-hospital neurological complications  HR (95% CI) |
| --- | --- | --- |
| Sex: Male | 1.53 (1.39 to 1.69) | 1.42 (1.24 to 1.65) |
| Hospital length of stay (+ 7 days) | 1.00 (0.97 to 1.03) | 1.08 (1.01 to 1.15) |
| Year of acute COVID-19 illness: 2021 | 1.06 (0.94 to 1.19) | 1.60 (1.23 to 2.03) |
| ICU admission: Yes | 0.68 (0.60 to 0.77) | 0.97 (0.80 to 1.18) |
| Neurological complications: Yes | - | 1.11 (0.52 to 2.37) |
| Antivirals: Yes |  | 0.93 (0.77 to 1.12) |
| Corticosteroids: Yes |  | 0.93 (0.79 to 1.09) |

Where HR: Hazard ratio; CI: Confidence interval; ICU: Intensive-Care-Unit; COVID-19: Coronavirus Disease-2019

**Supplemental Table 7** Observed prevalence and median time to symptom resolution stratified by reported in-hospital neurological complications

| Estimate | All | Female | Male |
| --- | --- | --- | --- |
| One or more in-hospital neurological complications ^a^ | | | |
| Reported cases | 12/27 | 6/11 | 6/16 |
| Crude prevalence | 44.4 (23.0 to 77.6) | 54.4 (20.0 to 118.7) | 37.5 (13.8 to 81.6) |
| Median time to resolution, months | 1.0 (0.3 to 2.3) | 2.1 (0.50 to 10.4) | 0.6 (0.15 to 1.9) |
| No in-hospital neurological complications, all survey participants | | | |
| Reported cases | 1,275/2,493 | 616/1,038 | 659/1,455 |
| Crude prevalence | 51.1 (48.3 to 54.0) | 59.3 (54.8 to 64.2) | 45.3 (41.9 to 48.9) |
| Median time to resolution, months | 2.7 (2.5 to 2.9) | 2.5 (2.3 to 2.8) | 1.9 (1.7 to 2.1) |
| No in-hospital neurological complications; matched ^b^ | | | |
| Reported cases | 678/1,214 | 292/444 | 386/770 |
| Crude prevalence | 55.8 (50.7 to 58.1) | 65.8 (58.4 to 73.8) | 50.1 (45.3 to 55.4) |
| Median time to resolution, months | 2.1 (2.0 to 2.3) | 3.0 (2.7 to 3.4) | 2.5 (2.2 to 2.7) |

^a^ Stroke, Seizures and/or Meningitis/Encephalitis

^b^ Matched cohort based on age at hospitalization (±5 years), sex, calendar period, and presence of one or neurological symptoms at acute hospitalization (**Supplemental File 1**).

**Supplemental Figure 1****.** Distribution of survey follow-up times.


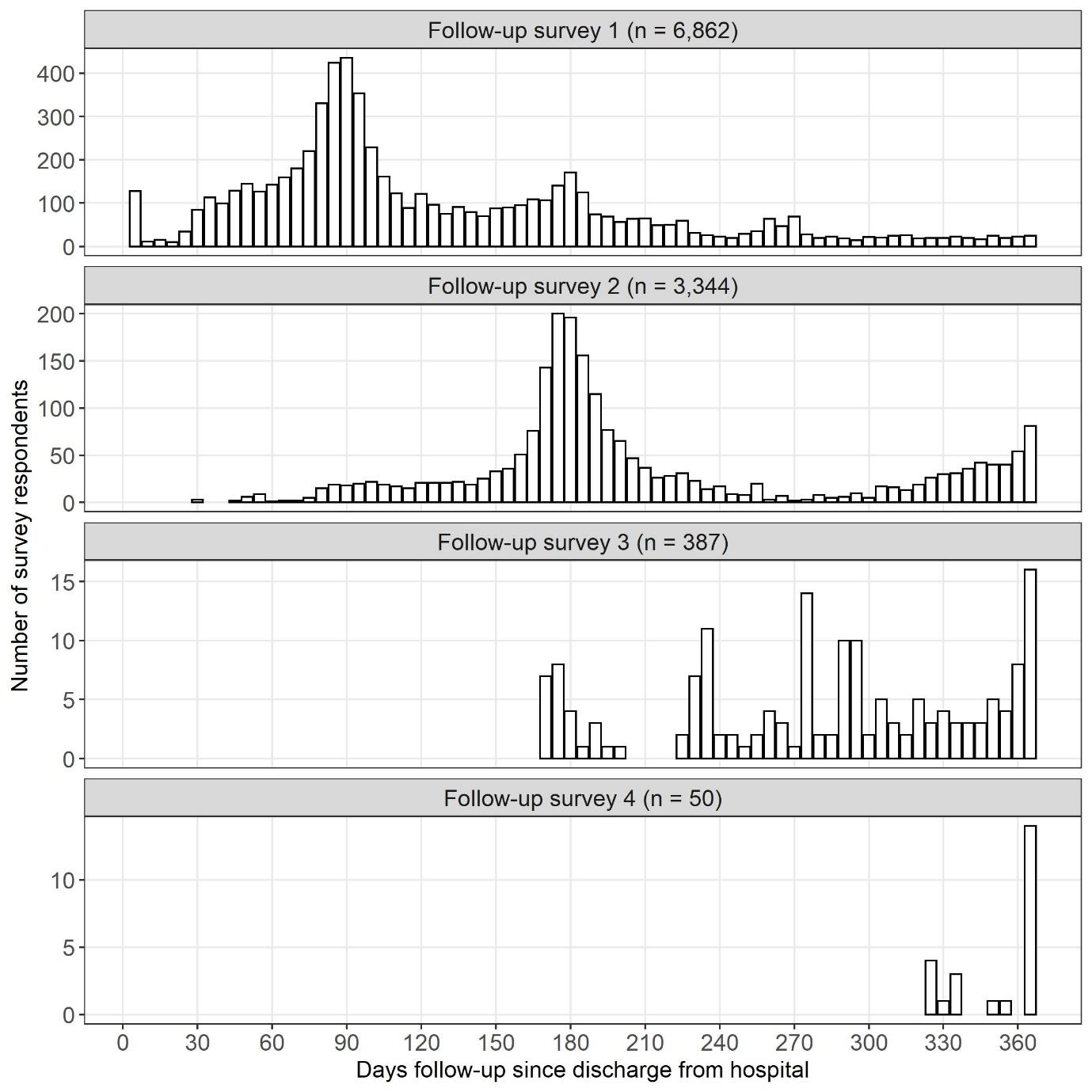


**Supplemental Figure 2.** Timing of acute COVID-19 hospitalization for all survey participants (n=6,862) evaluated for neurological symptoms and/or complications, frequencies are stratified by (A) Sex (B) ICU admission at any time during hospitalization.

**
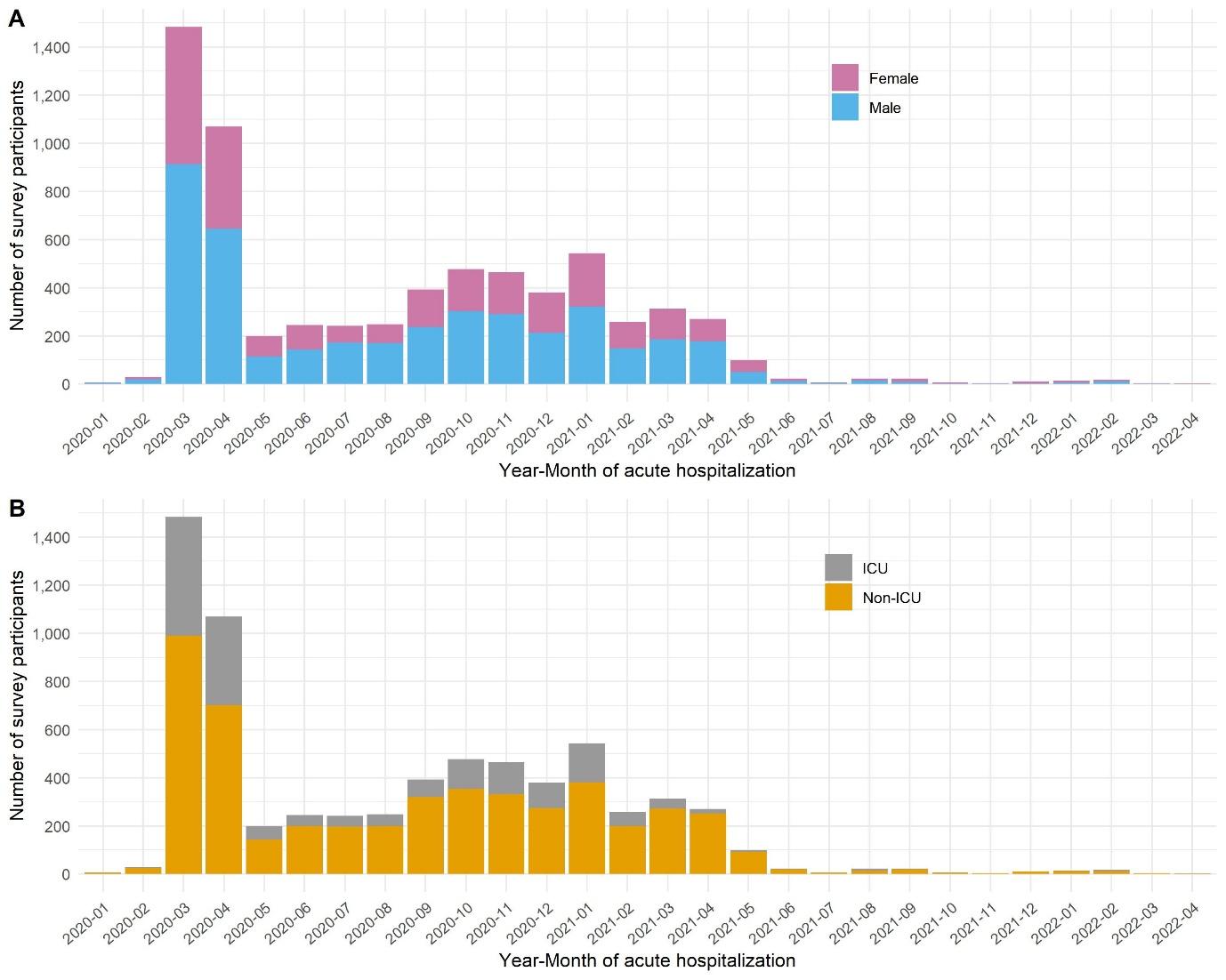
**

**Supplemental Figure 3:** Observed pairwise prevalence of neurological symptoms reported at hospital admission at the time of acute COVID-19.


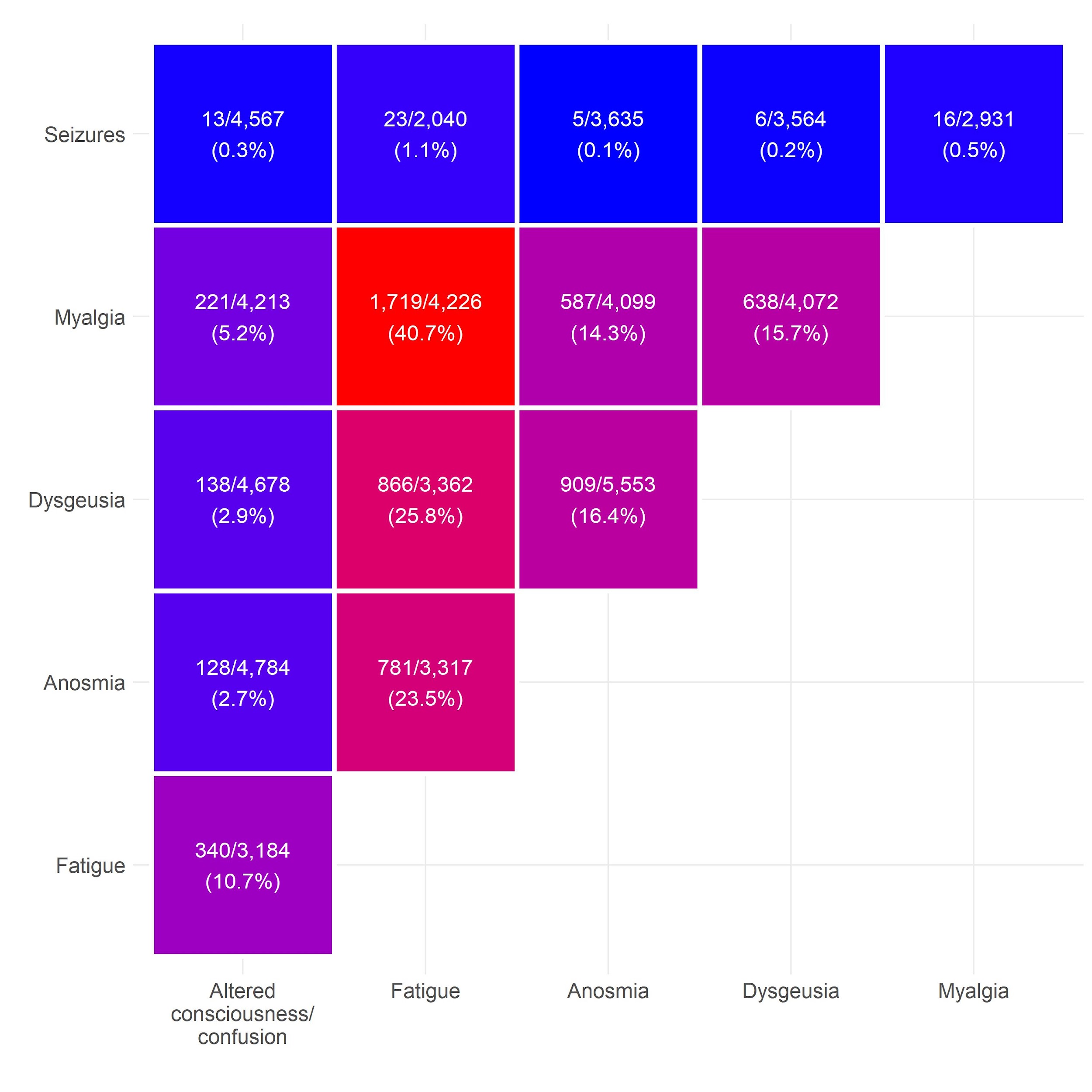


**Supplemental Figure 4:** Reported prevalence (%) of other neurological symptoms at initial follow-up survey.


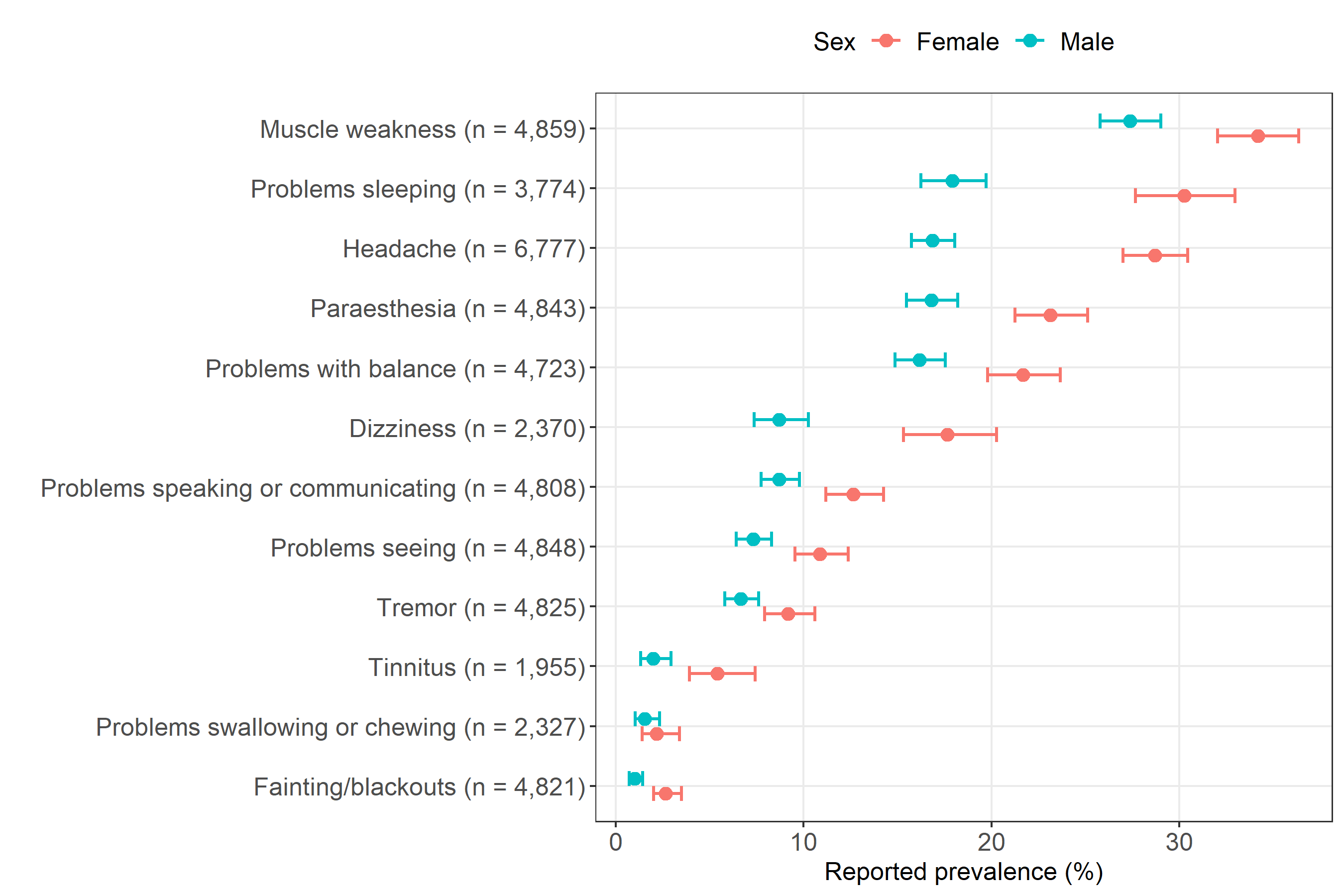


**Supplemental Figure 5A.** Median times to symptom resolution by age and sex, in months since acute hospital discharge: primary analysis. Sample sizes by symptom are 373 (Altered consciousness/confusion), 1,208 (Dysgeusia), 1,079 (Anosmia), 2,228 (Myalgia), 3,375 (Fatigue), 4,410 (One or more neurological symptoms)**.**


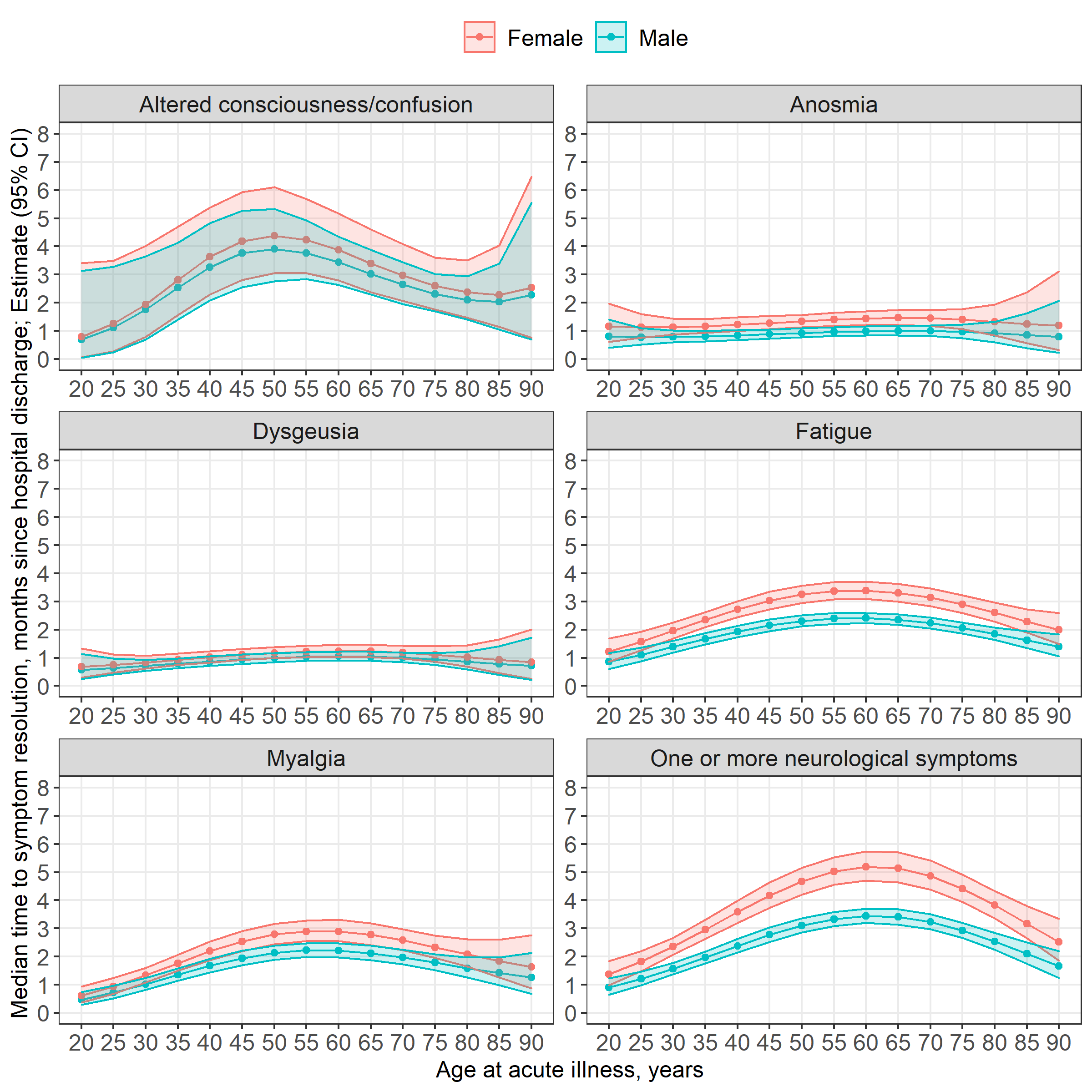


**Supplemental Figure 5B.** Median times to symptom resolution by age and sex, in months since acute hospital discharge: sensitivity analysis. Sample sizes by symptom are 373 (Altered consciousness/confusion), 1,208 (Dysgeusia), 1,079 (Anosmia), 2,228 (Myalgia), 3,375 (Fatigue), 4,410 (One or more neurological symptoms)**.**


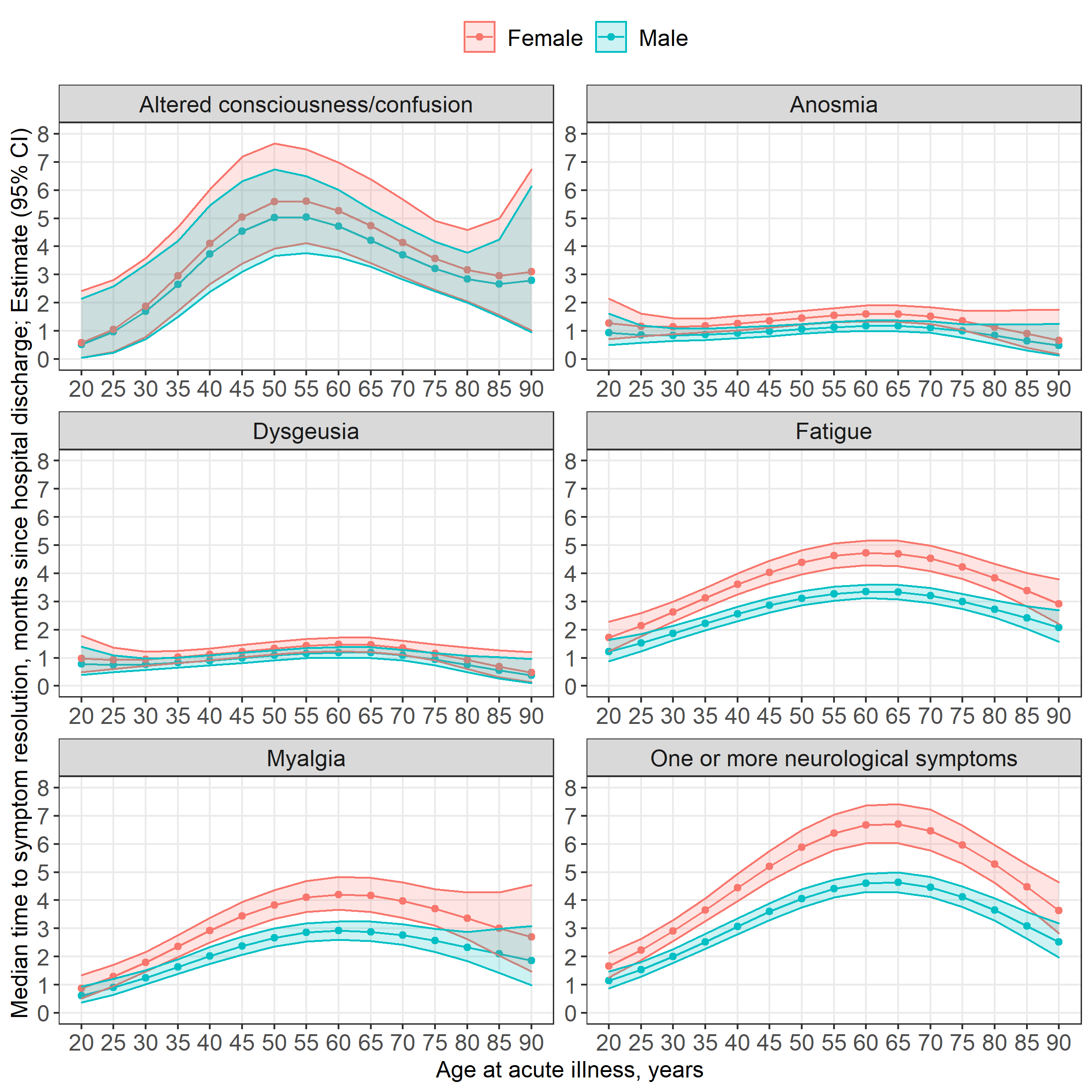


**Supplemental Figure 6:** Cumulative probabilities for time to symptom resolution for patients without (n = 896, left panel) and with neurological complications (meningitis/encephalitis, seizure or stroke) (n = 15; right panel) during hospital admission for acute COVID-19; primary analysis*. The left panel shows results for matched controls. **
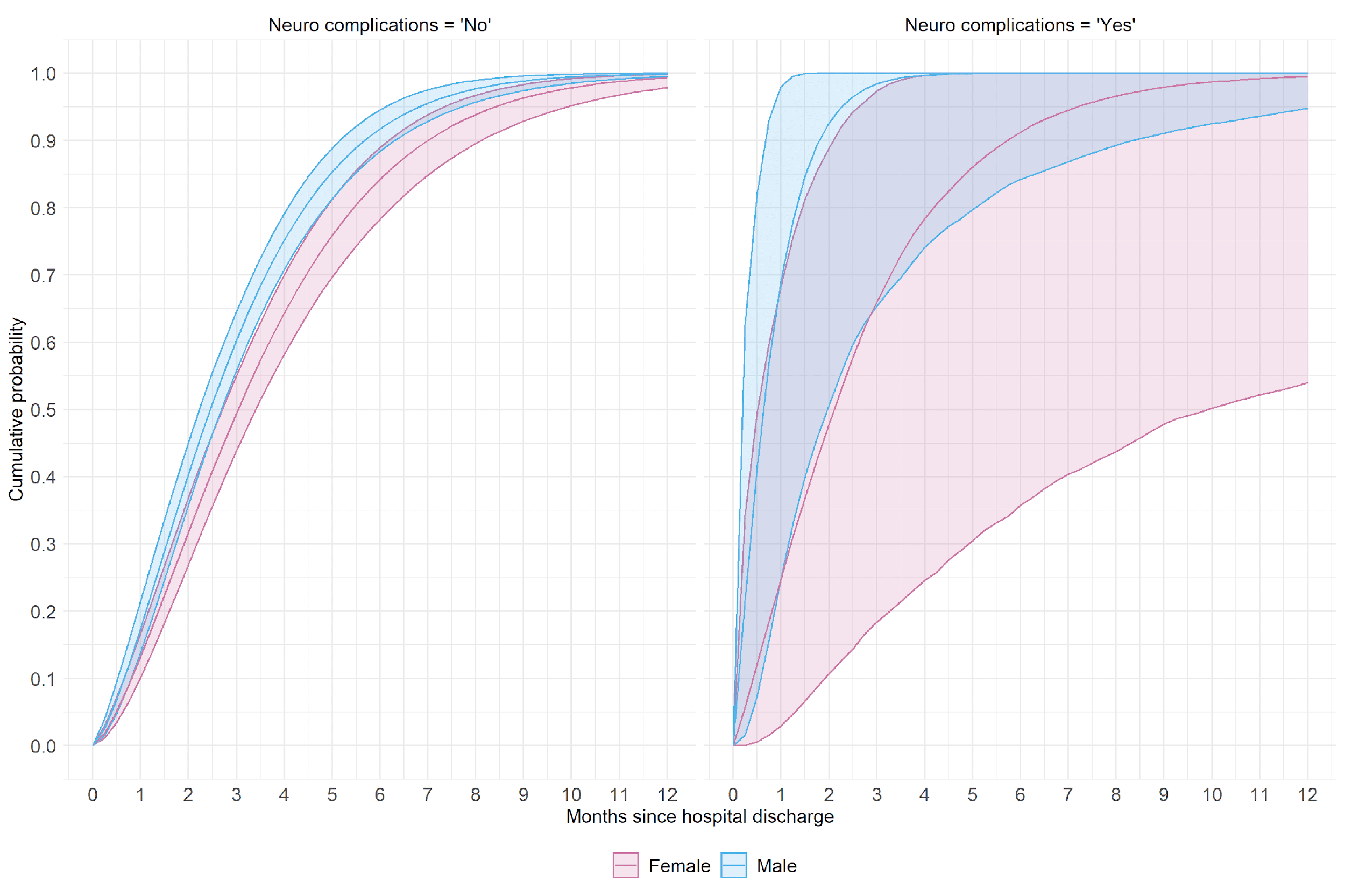
*** Sub-analysis of participants with complications data was also conducted, as shown below.

**Supplemental Figure 7.** Cumulative probabilities for time to symptom resolution, sensitivity analysis. Primary analysis is displayed in **Figure 2** (manuscript). Sample sizes by symptom are 373 (Altered consciousness/confusion), 1,208 (Dysgeusia), 1,079 (Anosmia), 2,228 (Myalgia), 3,375 (Fatigue), 4,410 (One or more neurological symptoms)**
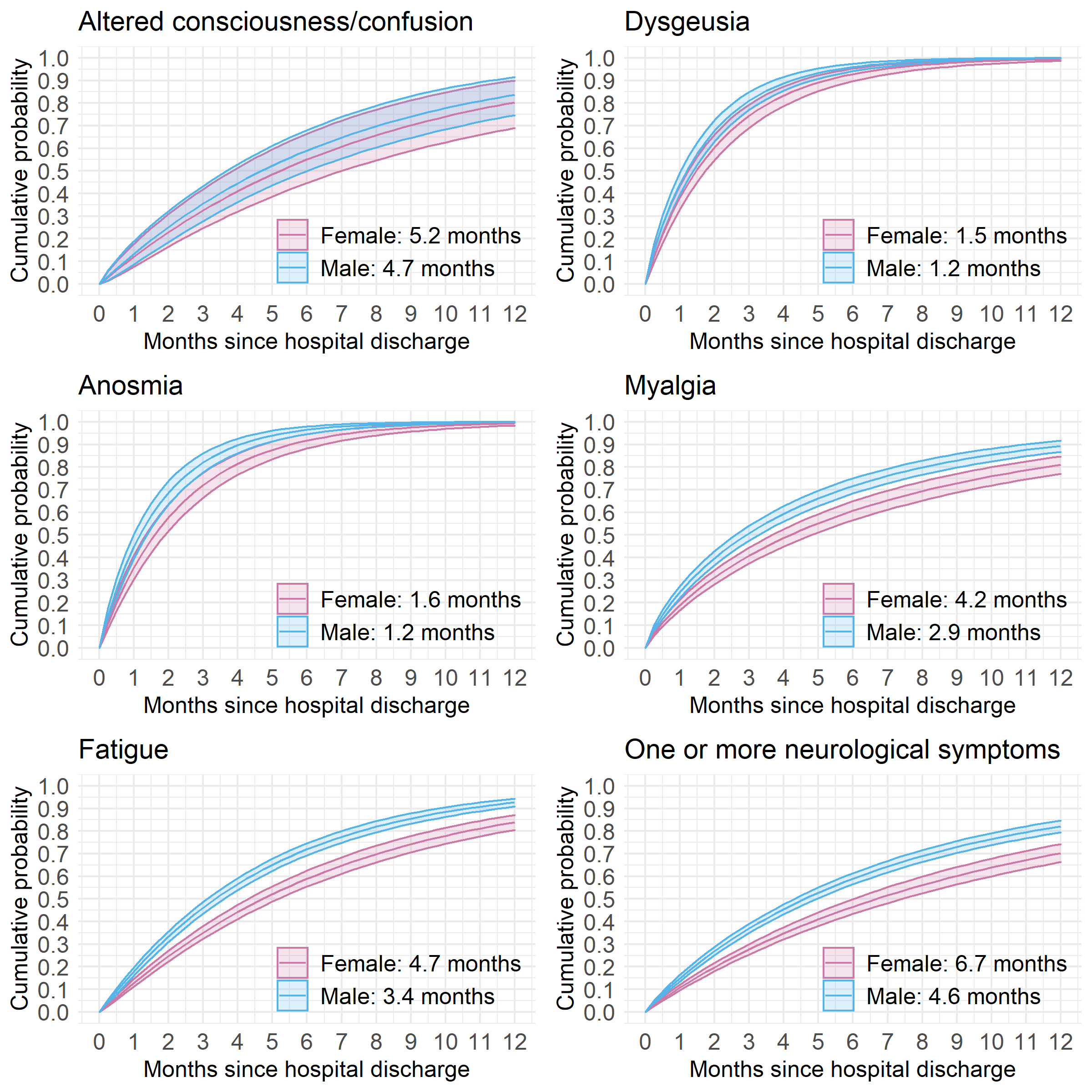
**

See **Supplemental File 1** for details of sensitivity analysis.

**Supplemental Figure 8**: Cumulative participant loss to survey follow-up, months since acute COVID-19 hospital discharge (n=6,862). The probability of participant loss increases over time from the discharge.


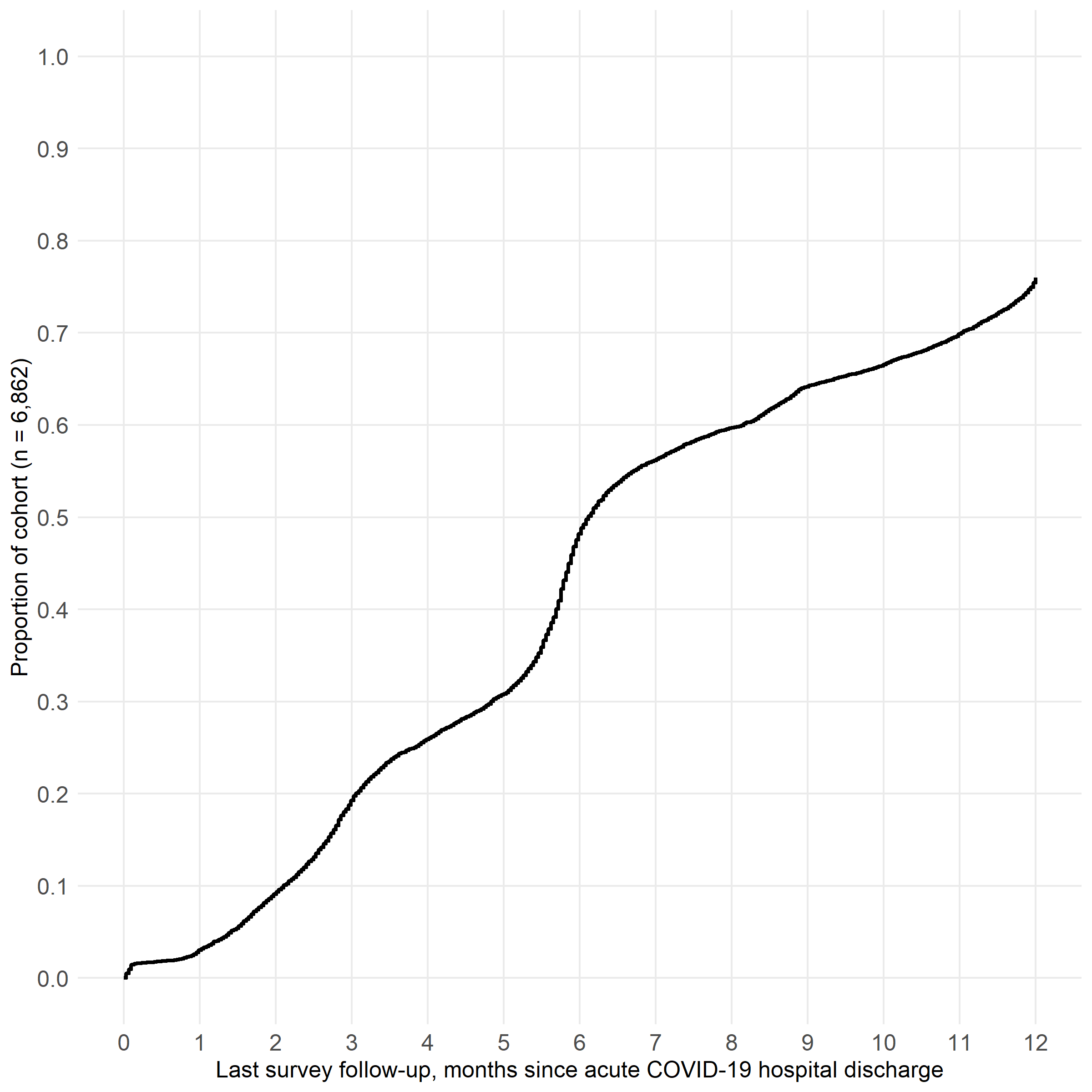

Supplement: fcae036_Supplementary_Data [file fcae036_supplementary_data.docx]
